# Telomerase is essential for cardiac differentiation and sustained metabolism of human cardiomyocytes

**DOI:** 10.1007/s00018-024-05239-7

**Published:** 2024-04-24

**Authors:** Shambhabi Chatterjee, Megan Leach-Mehrwald, Cheng-Kai Huang, Ke Xiao, Maximilian Fuchs, Mandy Otto, Dongchao Lu, Vinh Dang, Thomas Winkler, Cynthia E. Dunbar, Thomas Thum, Christian Bär

**Affiliations:** 1 Institute of Molecular and Translational Therapeutic Strategies (IMTTS), Hannover Medical School, Hannover, Germany; 2 Center of Translational Regenerative Medicine, Hannover Medical School, Hannover, Germany; 3 Fraunhofer Institute for Toxicology and Experimental Medicine, Hannover, Germany; 4 Translational Stem Cell Biology Branch, National Heart, Lung, and Blood Institute, National Institutes of Health, Bethesda, MD USA

**Keywords:** Telomerase, Telomere length, iPSC, CRISPR/Cas9, Cardiomyocytes, Single cell sequencing, Disease modelling

## Abstract

Telomeres as the protective ends of linear chromosomes, are synthesized by the enzyme telomerase (TERT). Critically short telomeres essentially contribute to aging-related diseases and are associated with a broad spectrum of disorders known as telomeropathies. In cardiomyocytes, telomere length is strongly correlated with cardiomyopathies but it remains ambiguous whether short telomeres are the cause or the result of the disease. In this study, we employed an inducible CRISPRi human induced pluripotent stem cell (hiPSC) line to silence TERT expression enabling the generation of hiPSCs and hiPSC-derived cardiomyocytes with long and short telomeres. Reduced telomerase activity and shorter telomere lengths of hiPSCs induced global transcriptomic changes associated with cardiac developmental pathways. Consequently, the differentiation potential towards cardiomyocytes was strongly impaired and single cell RNA sequencing revealed a shift towards a more smooth muscle cell like identity in the cells with the shortest telomeres. Poor cardiomyocyte function and increased sensitivity to stress directly correlated with the extent of telomere shortening. Collectively our data demonstrates a TERT dependent cardiomyogenic differentiation defect, highlighting the CRISPRi TERT hiPSCs model as a powerful platform to study the mechanisms and consequences of short telomeres in the heart and also in the context of telomeropathies.

**Supplementary Information:**

The online version contains supplementary material available at 10.1007/s00018-024-05239-7.

## Introduction

Aging is a non-modifiable risk factor which renders the heart more susceptible to stress [1]. Thus the global elderly population remains at a higher risk of developing heart failure [2]. Telomere shortening has been associated with aging and several health ailments. In addition to tissues or organs with high proliferation index, short telomeres have been also considered as risk factor for cardiovascular disorders (CVDs) [3–5]. Telomeres are hexameric DNA repeats (TTAGGG) encapsulated by shelterin proteins which together protect the ends of mammalian chromosomes. The enzyme telomerase, an RNA dependent DNA polymerase, synthesizes telomeric sequences using the telomere RNA component (TERC or TR) as template and the catalytic subunit telomerase reverse transcriptase (TERT). TERT expression is significantly downregulated in most tissues after birth, resulting in the progressive shortening of telomeres throughout life, which is considered a key driver of aging [6]. Indeed, at a certain critical telomere length, chromosomes become unprotected which induces genomic instability and subsequently leads to cell and tissue senescence.

The deleterious effects of telomere shortening can be counteracted by forced expression of TERT [7]. Systemic reactivation of telomerase by means of AAV9-TERT gene therapy extend the life span of mice even if applied in adult and aged mice [8]. Importantly, when applied in an aging-relevant disease model, AAV9-TERT therapy was shown to be cardioprotective in a myocardial infarction mouse model [9]. Telomerase has indeed emerged as a potential therapeutic target to prevent heart failure, which is mediated through both telomeric and non-telomeric functions of TERT [10–12]. Sahin et al. reported that telomere dysfunction is detrimental to mitochondrial metabolism via the telomere-p53-PGC axis, which ultimately disrupts cardiac function [13]. In line with this, Mourkioti et al. reported that short telomeres in a Duchenne muscular dystrophy mouse model predisposed to severe heart failure in contrast to mice with normal telomere length which exhibit only minimal cardiac dysfunction [14]. In a human context, we have demonstrated that leukocyte telomere lengths in patients with hypertrophic cardiomyopathy inversely correlates with disease severity [3]. These observations have contributed to the debate of whether short telomeres give rise to CVDs or if CVDs induce telomere attrition in impaired cardiomyocytes.

A testing platform to answer this question and to delineate the complex interplay of canonical or non-canonical telomerase function and telomere length dynamics in CVDs is missing to date. Human induced pluripotent stem cells (hiPSCs) as well as gene editing technologies such as CRISPR (clustered regularly interspaced short palindromic repeats) have gained extensive popularity in the field of cardiovascular disease modeling and drug discovery. We here developed a CRISPR interference (CRISPRi) model for an inducible and reversible silencing of TERT in hiPSCs. Consecutive passaging of these cells in the presence or absence of TERT permitted the generation of hiPSC with long and short telomeres. These cells could be further differentiated into cardiomyocytes with long and short telomeres to investigate the consequences of telomere length on cardiac differentiation capacity and response to stress stimuli in the resulting cardiomyocytes. This novel in vitro system for telomere modulation represents an excellent platform to investigate both telomere biology in cardiomyocytes and potential mechanisms of telomere driven CVDs.

## Results

### In vitro modelling of telomerase in hiPSCs using CRISPRi

HiPSCs are known to possess long telomeres maintained primarily by high telomerase activity. We tested if engineered down-modulation of TERT would lead to replicative telomere shortening at the hiPSC stage to produce hiPSCs and subsequently hiPSC-cardiomyocytes with long and short telomeres. To do so, we applied an inducible CRISPRi technology at the hiPSC stage to inhibit telomerase expression and activity [15]. The inducible CRISPRi TERT hiPSCs contained a gRNA targeted to the TERT promoter along with a deactivated Cas9 fused to a KRAB repression domain, allowing inducible tuning of TERT gene expression via doxycycline exposure. As hiPSCs exhibit high proliferation rates we anticipated that long telomeres would shorten with subsequent passages in presence of doxycycline induction which silenced TERT, allowing tunable modulation of telomere lengths.

**Fig. 1:**
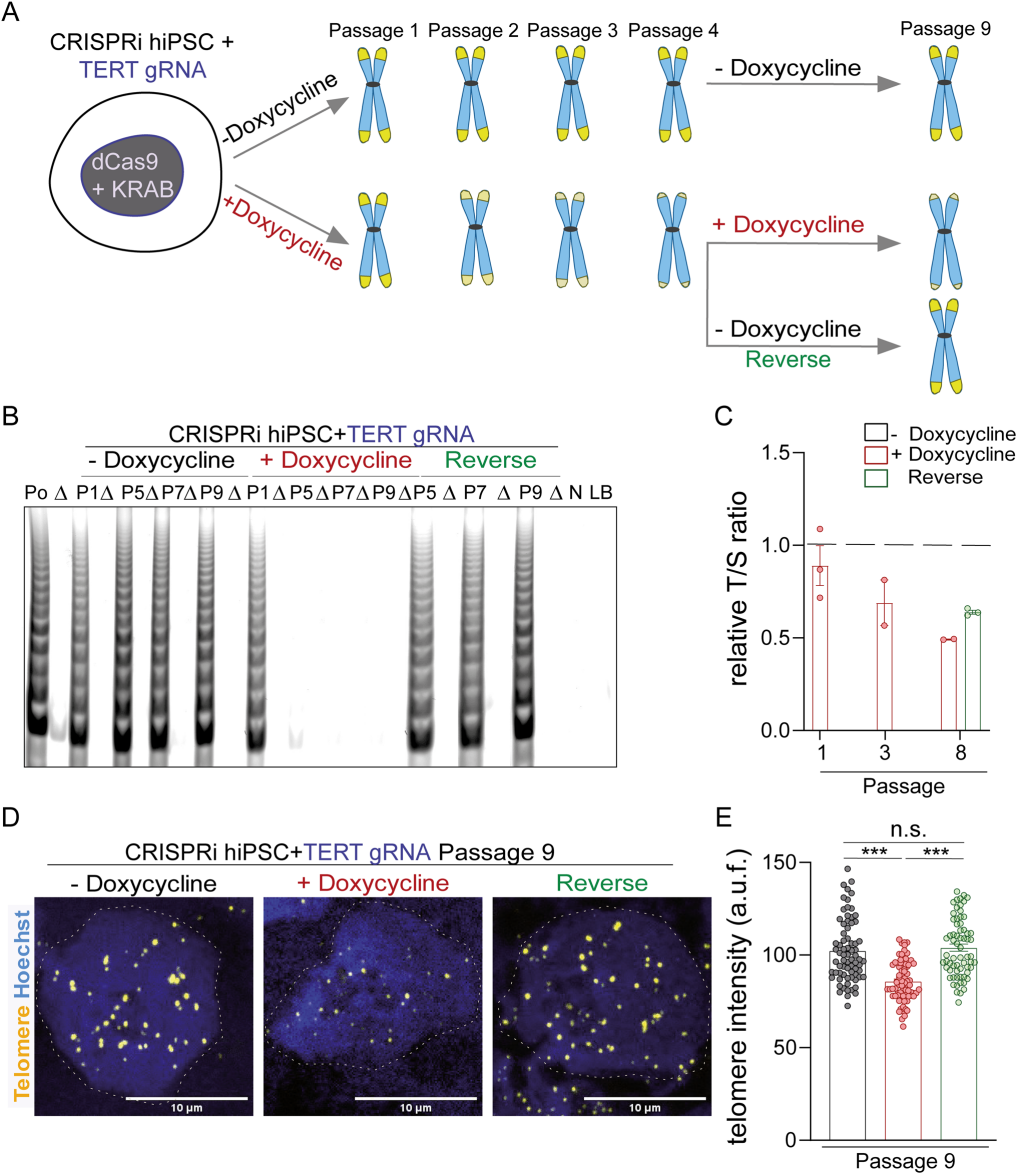
Modulation of telomerase and telomere lengths in hiPSCs using CRISPRi. **A** Schematic representation of the telomerase modulation in the CRISPRi TERT hiPSCs using doxycycline induction and reversal of telomerase modulation by withdrawal of doxycycline. The replicative shortening (and recovery in reverse experiment strategy) of telomere length is expected to occur gradually through subsequent passaging of the cells. **B** Telomerase activity in cell lysates of CRISPRi TERT hiPSCs under doxycycline treatment was negligible from passage 5 (+ Doxycycline), which could be rescued back to untreated (− Doxycycline) control levels after removal of doxycycline treatment. (n = 200,000 cells per group), Po indicates cell lysate of TRAP positive sample (hiPSCs); ∆ indicates heat inactivated cell lysate; P indicates passage of the hiPSCs; N indicates cell lysate of TRAP negative sample (HUVEC); LB indicates lysis buffer. **C** The fold change in telomere lengths of CRISPRi TERT hiPSCs as measured by qPCR. Data shows reduced telomere lengths after CRISPRi induction (+ Doxycycline, red bar) and further rescue upon removal of doxycycline induction (Reverse, green bar). The dotted black line indicates telomere length of the control (− Doxycycline) CRISPRi TERT iPSCs. (n = 2–3 sample per group). **D**–**E** Representative images and quantification performed for CRISPRi TERT hiPSCs at passage 9 using TEL-qFISH. Shortening of telomere lengths is observed upon doxycycline-mediated CRISPRi induction (+ Doxycycline, red bar) which is reversed after removal of doxycycline (Reverse, green bar) (n ≥ 66 nuclei per group). Scale bar indicates 10 µm; The dotted grey line indicates the nuclei region used for qFISH analysis. hiPSCs = human induced pluripotent stem cells; *** p < 0.001; One-way ANOVA, Kruskal–Wallis test with Dunn’s multiple comparison test

CRISPRi targeting TERT was induced in one set of hiPSCs with 3 µM doxycycline (+ Doxycycline) and a parallel untreated group (- Doxycycline) were maintained as controls (Fig. 1A). An additional reversal group was examined, where doxycycline treatment was removed from the hiPSCs after 4 passages (Reverse). The telomerase activity in hiPSCs was gradually shut down in the presence of 3 µM doxycycline (Supplementary Fig. 1A, B). Complete loss of telomerase activity was confirmed by the telomere repeat amplification protocol (TRAP) assay as early as passage 5, which could be fully restored within 4 weeks of doxycycline removal at passage 9 (Fig. 1B). In line with this, replication-associated reduction in telomere lengths was observed by TEL-qPCR, with re-extension once doxycycline induction of CRISPRi was stopped (Fig. 1C). The loss in telomere length was validated by TEL-qFISH following the 9th passage under continuous doxycycline treatment, as well as rescue after 4 additional passages following doxycycline removal (Fig. 1D, E). Together, these data demonstrate that CRISPRi targeting the TERT promoter can be used to modulate telomerase activity and telomere length in hiPSCs. Hence, this system allowed us to develop a series of hiPS cell populations with different telomere lengths.

### HiPSC with short telomeres exhibit impaired cardiomyocyte differentiation

Our next objective was to generate hiPSC-cardiomyocytes with long and short telomeres. We analyzed CRISPRi TERT hiPSC colonies with high (no doxycycline), intermediate (doxycycline for two passages) (Supplemental Fig. 1A, B), and negligible (doxycycline for five passages) telomerase activity (Fig. 1B), and confirmed replicative shortening of telomeres within these passages (Supplemental Fig. 1C, D). To rule out that a reduction in telomerase activity and telomere length impacted hiPSC stemness per se, we performed immunostaining for the stem cell markers SSEA4 and TRA-1-60, and found no changes in protein levels in hiPSCs at doxycycline passage 5 (short telomeres; + Doxycycline) compared to doxycycline untreated cells (long telomeres;− Doxycycline) (Supplemental Fig. 1E). Trilineage differentiation assays demonstrated that CRISPRi TERT hiPSCs with short telomeres following five passages in doxycycline retained the potential to differentiate into all three germ layers (Supplemental Fig. 1F).

**Fig. 2:**
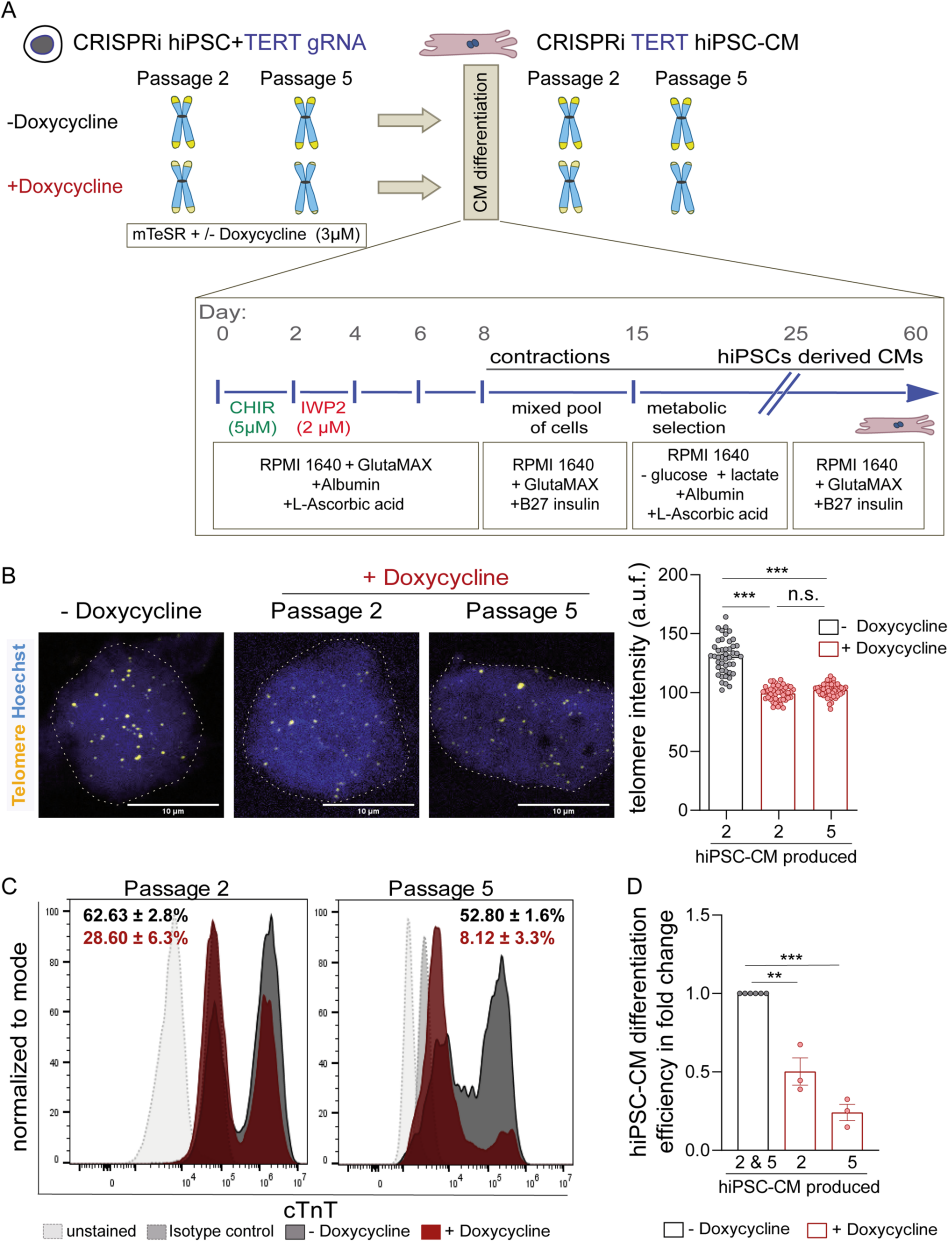
Cardiomyocyte differentiation capacity depends on telomerase activity of the stem cells. **A** Schematic describing the proposed in vitro model to generate hiPSC-cardiomyocytes with long and short telomeres. Schematic also depicts the CRISPRi TERT hiPSC maintenance and differentiation protocol used in the study. CRISPRi TERT hiPSCs treated with doxycycline possess shorter telomeres and should result in hiPSC-cardiomyocytes with shorter telomere lengths as opposed to the untreated CRISPRi TERT hiPSCs. **B** Telomere length measured via TEL-qFISH in hiPSC-cardiomyocytes arising from CRISPRi TERT hiPSCs with long (− Doxycycline) and short telomeres (passage 2 and passage 5 + Doxycycline, red bars) (n ≥ 45 nuclei per group). The dotted grey line indicates the nuclei region used for qFISH analysis. **C** HiPSC-cardiomyocyte differentiation efficiency measured by cTnT expression via flow cytometry; at day 12 of the cardiomyocyte differentiation protocol; from CRISPRi hiPSCs (with and without doxycycline treatment until passage 2 and 5) (n ≥ 5000 events per sample). Isotype controls (grey dotted line, filled) and unstained cells (light grey dotted line, filled) were used as controls. CRISPRi TERT hiPSC-cardiomyocytes arising from doxycycline treated hiPSCs are depicted in red and the ones arising from control hiPSCs (− Doxycycline) are depicted in dark grey. Representative plots with mean ± SEM are depicted. **D** Quantification of hiPSC-cardiomyocyte differentiation efficiency amongst the CRISPRi hiPSCs with and without doxycycline treatment is represented in terms of fold change. All data are mean fold change relative to control ± SEM (n = 3 independent differentiation experiments); hiPSC-CM = human induced pluripotent stem cell derived cardiomyocyte; *p < 0.05; **p < 0.01; ***p < 0.001; One-way ANOVA, Tukey multiple-comparisons test

We then chose CRISPRi TERT hiPSCs from passages 2 (intermediate telomerase activity and telomere length) and 5 (negligible telomerase activity and short telomeres) to evaluate whether telomerase activity and telomere length influence cardiomyocyte differentiation and function. To do so, the hiPSCs were differentiated into cardiomyocytes via the well-established Wnt-pathway modulation protocol [10, 16] (Fig. 2A). Since we previously established that telomerase is rapidly silenced during the differentiation of hiPSCs into cardiomyocytes [10] and that reversal of telomerase activity requires 1 passage (i.e*.* 5–7 days, Fig. 1B), doxycycline treatment was discontinued once the differentiation protocol was initiated (Fig. 2A). The CRISPRi TERT hiPSC-cardiomyocytes generated under each condition were purified using a metabolic selection [17] and then cultured until day 60 for maturation (Fig. 2A). We measured the telomere lengths in the purified cardiomyocytes via TEL-qFISH, and confirmed that the differentiated hiPSC-cardiomyocytes at passage 2 retained the telomere length variation seen at the hiPSC stage (Fig. 2B). However, no further reduction in telomere lengths was observed in CRISPRi TERT hiPSC-cardiomyocytes arising from passage 5 relative to passage 2 cells at day 60 (Fig. 2B), suggesting that hiPSCs with shorter telomeres and lack of TERT expression do not differentiate efficiently into cardiomyocytes or that those cardiomyocytes are lost during metabolic selection.

To test this, we studied morphological changes by brightfield microscopy during differentiation and observed marked qualitative differences in the potential of CRISPRi TERT induced hiPSCs to generate cardiomyocytes (Supplemental Videos 1 and 2). To confirm these observations, cells were collected at day 12 of the hiPSC-cardiomyocyte differentiation protocol, *i.e.* prior to metabolic selection, and analyzed for cardiac Troponin T (cTnT) expression via flow cytometry (Fig. 2C). The gene TNNT2 encodes for cTnT which is expressed at the embryonal stages and throughout the process of cardiomyogenesis. In the adult heart, cTnT expression is crucial for the contractile function of the cardiomyocytes [18]. The control hiPSCs generated 50–60% cTnT positive cells (Fig. 2C, dark grey histogram plots). Notably, as the telomerase activity reduced and telomeres shortened, the hiPSCs lost their ability to differentiate into cardiomyocytes as determined by flow cytometry analysis, with ~ 30% cTnT positive cells from passage 2 of doxycycline-treated hiPSCs (Fig. 2C, red histogram plot on the left side) and only ~ 8% cTnT positive cells from passage 5 doxycycline-treated hiPSCs (Fig. 2C, red histogram plot on the right side). Thus, suppression of TERT in the hiPSCs stage leads to a severe decrease in hiPSC-cardiomyocyte differentiation potential (Fig. 2C, D).

### Short telomeres lead to profound transcriptome changes associated with developmental pathways

**Fig. 3:**
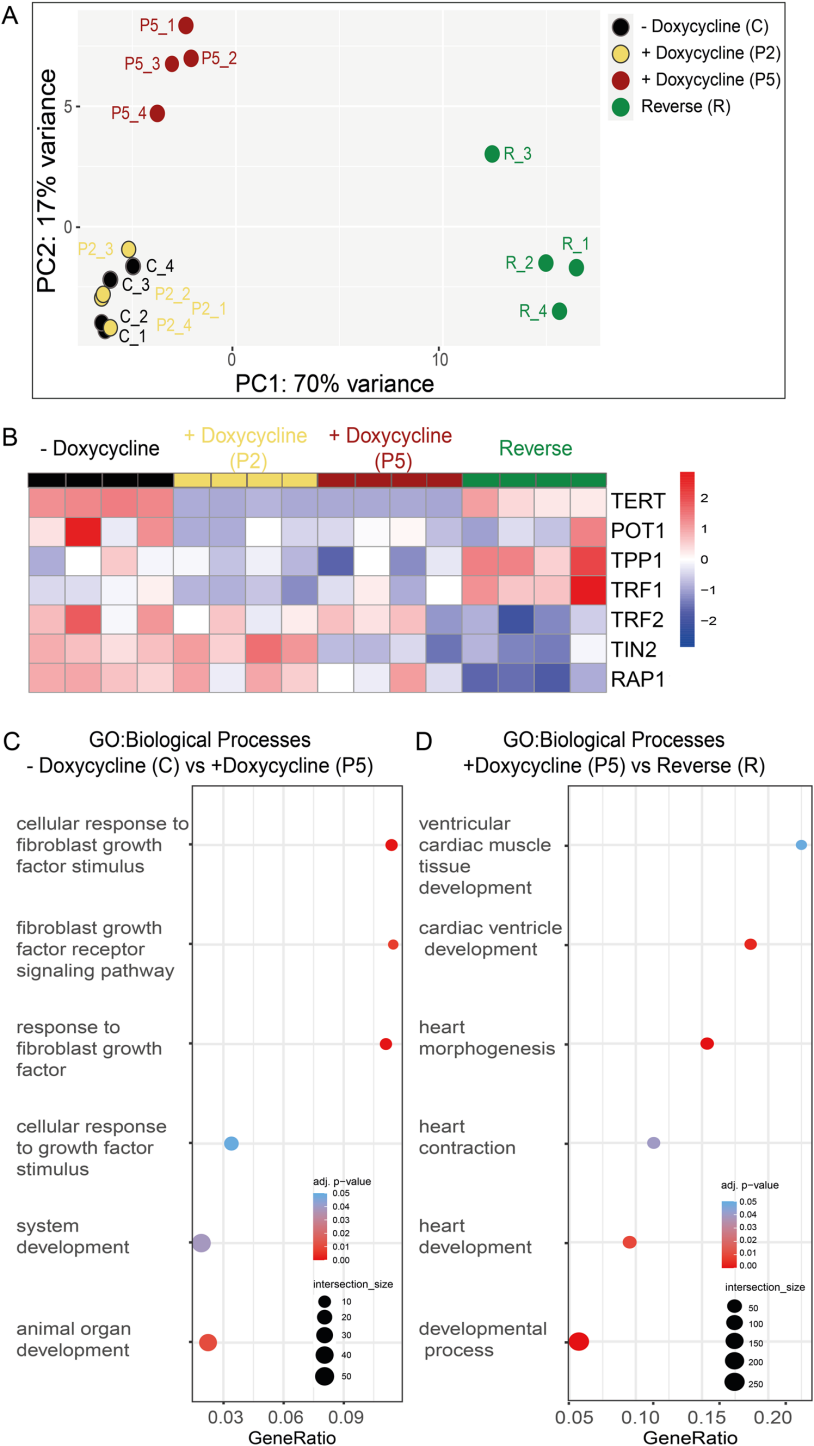
Global transcriptomic changes after telomerase modulation in CRISPRi TERT hiPSCs. **A** PCA plot indicates the distance between CRISPRi TERT hiPSC samples. X and Y axis show principal component 1 and principal component 2 that explain 70% and 17% of the total variance, respectively. (n = 4 per condition) CRISPRi TERT hiPSCs long (− Doxycycline, **C**, black circles), intermediate (+ Doxycycline, P2, yellow circles), short telomeres (+ Doxycycline, P5, red circles) and the reverse group (doxycycline removed for 2 weeks from P5 samples, R, green circles) (Supplemental Table 1 for list of top 2000 highly expressed genes) **B** Heatmap illustrates the differential gene expression of telomerase and telomere shelterin proteins between CRISPRi TERT hiPSCs under different conditions (x-axis: C, P2, P5 and R) (n = 4 /condition) in red: upregulation, in blue: downregulation **C** Bubble plots of functional enrichment analysis. The plot shows selected Gene Ontology Biological Processes enriched after treatment with doxycycline 5 passages. (Supplemental Table 2 for whole functional enrichment analysis). **D** Gene Ontology (GO) analysis of differentially regulated genes shows overrepresented biological process between R and P5 as depicted in bubble plots. Bubble size represents the ratio between the genes connected to a process and the overall query size. The color scale shows the adjusted p-value (Supplemental Table 3 for whole functional enrichment analysis)

To discern how telomerase/telomere modulation at the hiPSC stage could influence cardiomyogenesis, we performed bulk RNA sequencing (RNA-seq) from the CRISPRi TERT hiPSCs under various doxycycline treatment conditions. Untreated and doxycycline treated (passages 2 and 5) CRISPRi TERT hiPSCs as well as the reversal group (doxycycline removed for 2 weeks after 5 passages doxycycline) were investigated. Principal Component Analysis (PCA) revealed a distinct clustering for the samples with the shortest telomeres and the reversal group, while untreated samples and samples with moderate telomere shortening cluster closely together (Fig. 3A, Supplemental Fig. 2A and Supplemental Table 1). As expected, TERT expression was significantly downregulated in the doxycycline groups and re-expressed in the reversal group (Fig. 3B) which further validated our in vitro model of telomerase modulation. The genes encoding for shelterin proteins were also found to be significantly altered between the groups (Fig. 3B). Severe telomere shortening (5 passages doxycycline) introduced profound changes in biological pathways related to development processes and more specifically in response to fibroblast growth factor (Fig. 3C, Supplemental Fig. 2B, Supplemental Table 2). Strikingly, when telomerase expression was reinstated, cardiomyogenesis related genes and pathways were highly upregulated (Fig. 3D, Supplemental Fig. 2C and Supplemental Table 3).

**Fig. 4:**
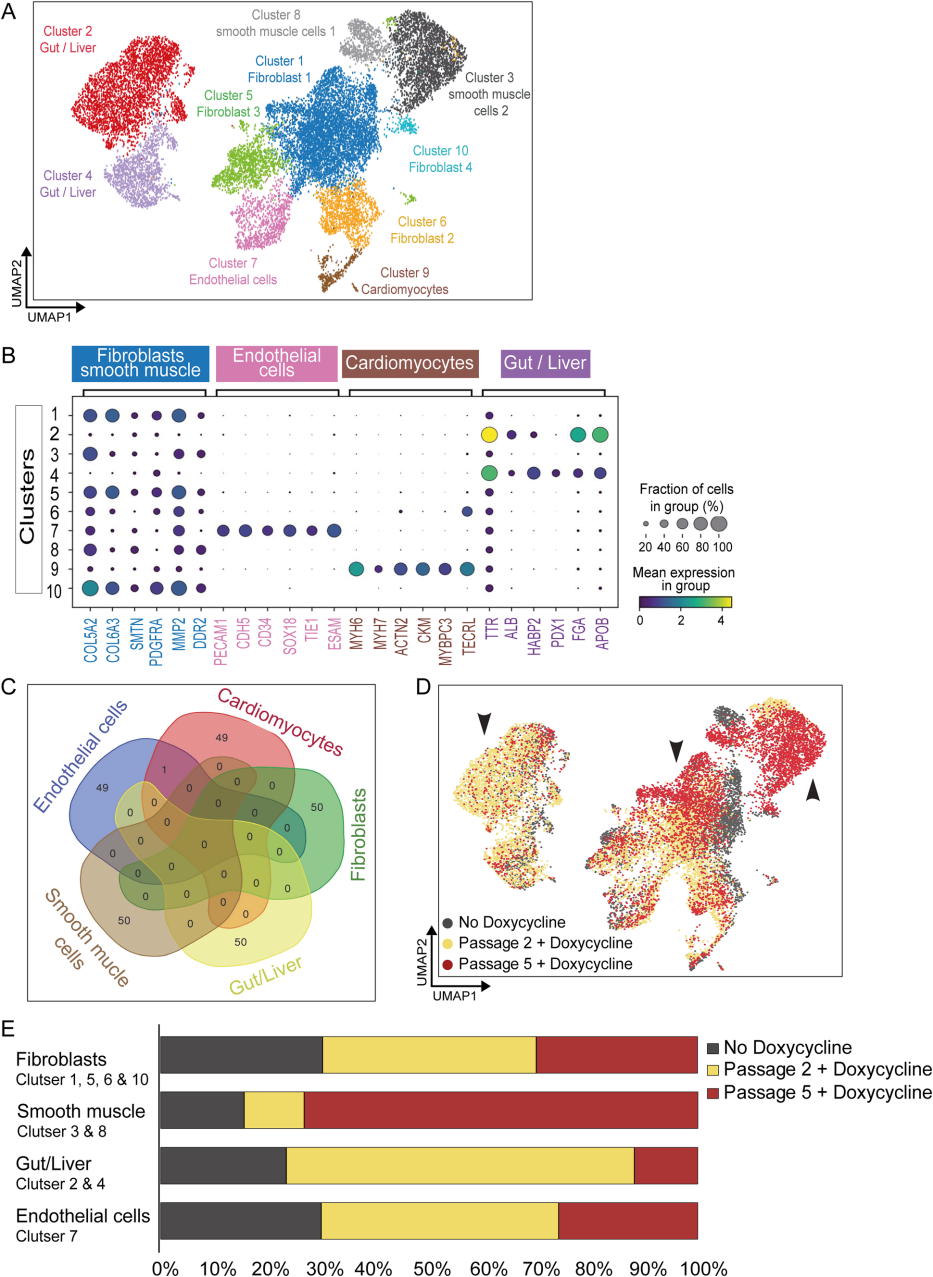
Mesodermal differentiation is influenced by telomerase activity of the stem cells**. A** Identification of the 10 clusters after single cell RNA sequencing of day 12 differentiated cells arising from CRISPRi TERT hiPSCs with long (− Doxycycline) and short (+ Doxycycline for passage 2 and passage 5) telomere lengths, n =  > 4500 cells/group. **B** Gene expression profiles (columns) of selected 6 cell type-specific markers (well known from literature) used for categorizing each cluster into a particular mesodermal cell type as shown in the dot plot. Bar on the right displays the percentage of total dataset represented in every cluster, showing the abundance of each cell type found by clustering analysis. **C** Venn diagram depicting top 50 genes of the 5 main cell types (from the total 10 clusters). Only 1 gene overlaps between Endothelial and Cardiomyocyte cell cluster. **D** Distribution of the cells arising from differentiation of CRISPRi TERT hiPSCs with long telomeres (− Doxycycline, dark grey) and intermediate telomere length (two passage + Doxycycline, yellow) and short telomere length (five passage + Doxycycline, red) across the different clusters. Black arrows highlight shift in the cell distribution. **E** Abundance of cells in specific clusters arising from the various groups of the doxycycline induced CRISPRi TERT hiPSCs

We next investigated the nature of the other cell types arising during impaired cardiomyocyte differentiation. The presence of varied cell types or differentiation regions during the CRISPRi TERT hiPSC-cardiomyocyte differentiation with short telomeres can also be observed in the Supplemental Video 2 relative to Supplemental Video 1. CRISPRi TERT hiPSCs with high, intermediate and negligible telomerase activity and telomere length were differentiated, followed by collection of the cells at day 12 for single cell RNA-sequencing (scRNA-seq). Ten major clusters were observed (Fig. 4A). Analysis of the genes expressed in each cluster based on well-known marker genes from the literature resulted in the identification of clusters present in the differentiated pool of cells at day 12 (Fig. 4B). No overlap was observed between the top 50 highly expressed genes from each cluster (except one gene overlap between endothelial cells and cardiomyocytes), confirming the robustness of our cluster identification analysis (Fig. 4C). As expected, cells arising from the mesodermal lineage clustered into expected cardiomyocytes (cluster 9), endothelial cells (cluster 7), fibroblasts (cluster 1, 5 and 10), and smooth muscle cells (cluster 3 and 8) [19]. Diffusion map analyses were performed to map mesoderm differentiation trajectories, suggesting movement from clusters 1 & 3 (Fibroblasts) either towards cluster 9 (Cardiomyocytes) or clusters 2 and 4 (Gut/Liver) (Supplemental Fig. 3A).

Strikingly, two clusters (2 and 4) with gut and liver cell markers, were noted predominantly in the cells from passage 2 doxycycline-treated CRISPRi TERT hiPSCs (Fig. 4D, E—black arrow on the left side for the yellow dots; yellow bar on the plot). On further analysis, the cells with the shortest telomeres gave rise to more cells belonging to clusters 3, 8 and 10, identified as smooth muscle or fibroblast cells (Fig. 4D, E—black arrow on the middle and right side in the red dots, red bar on the plot). This was in line with our observation from the CRISPRi TERT hiPSCs RNA-seq data (Fig. 3C, Supplemental Fig. 2B) where fibroblast related gene signatures were upregulated in short telomere group. A list of top genes expressed from each telomere condition also highlighted abundance of the respective cell types in the specific clusters (Supplemental Fig. 3B). The differentiated cells arising from CRISPRi TERT hiPSCs with long telomeres (− Doxycycline) were equally distributed within all 10 clusters whereas the cells differentiated from hiPSCs with shorter telomeres cluster specifically in certain cell types (Fig. 4D, E and Supplemental Fig. 3B).

However, the proportion of cardiomyocytes (cluster 9) was underrepresented in all samples (Supplemental Fig. 3C), since cells with large cell diameters are not well captured during scRNA-seq performed using the 10X Genomics platform [20]. Nevertheless, we looked into highly expressed genes within the small proportion of cardiomyocytes that were sequenced, and observed a number of differentially expressed genes (Supplemental Fig. 3D). Notably, the expression of EMC10, FHL2, MTRNR2L8, MTRNR2L10, and SFRP1 correlated with the degree of telomere shortening (highlighted in green text, Supplemental Fig. 3D). Long telomere cardiomyocytes highly expressed SFRP1 whereas the cardiomyocytes possessing short telomeres expressed of the isoform SFRP3 (highlighted in red text, Supplemental Fig. 3D). This was striking as Secreted frizzled related protein (SFRP) family members are well known Wnt-antagonists as well as known to be involved with cardiac metabolic diseases [21, 22].

Taken together, the scRNA-seq indicates that suppressed TERT does not prevent hiPSCs from initiation of endo-mesodermal differentiation. However, further differentiation into cardiomyocytes is hampered in the absence of telomerase activity and sufficient telomere reserve at the hiPSC stage most likely attributed to global changes in gene expression which affect the Wnt signaling pathway. The pattern of cell clusters changes between the differentiated cells arising from hiPSCs with high, intermediate and negligible telomerase activity and gradually shortened telomere length. This implies that telomerase function is a crucial factor which determines cardiomyocyte differentiation capacity of hiPSCs.

### Short telomeres predispose hiPSC-cardiomyocytes to cell death under stress conditions

**Fig. 5:**
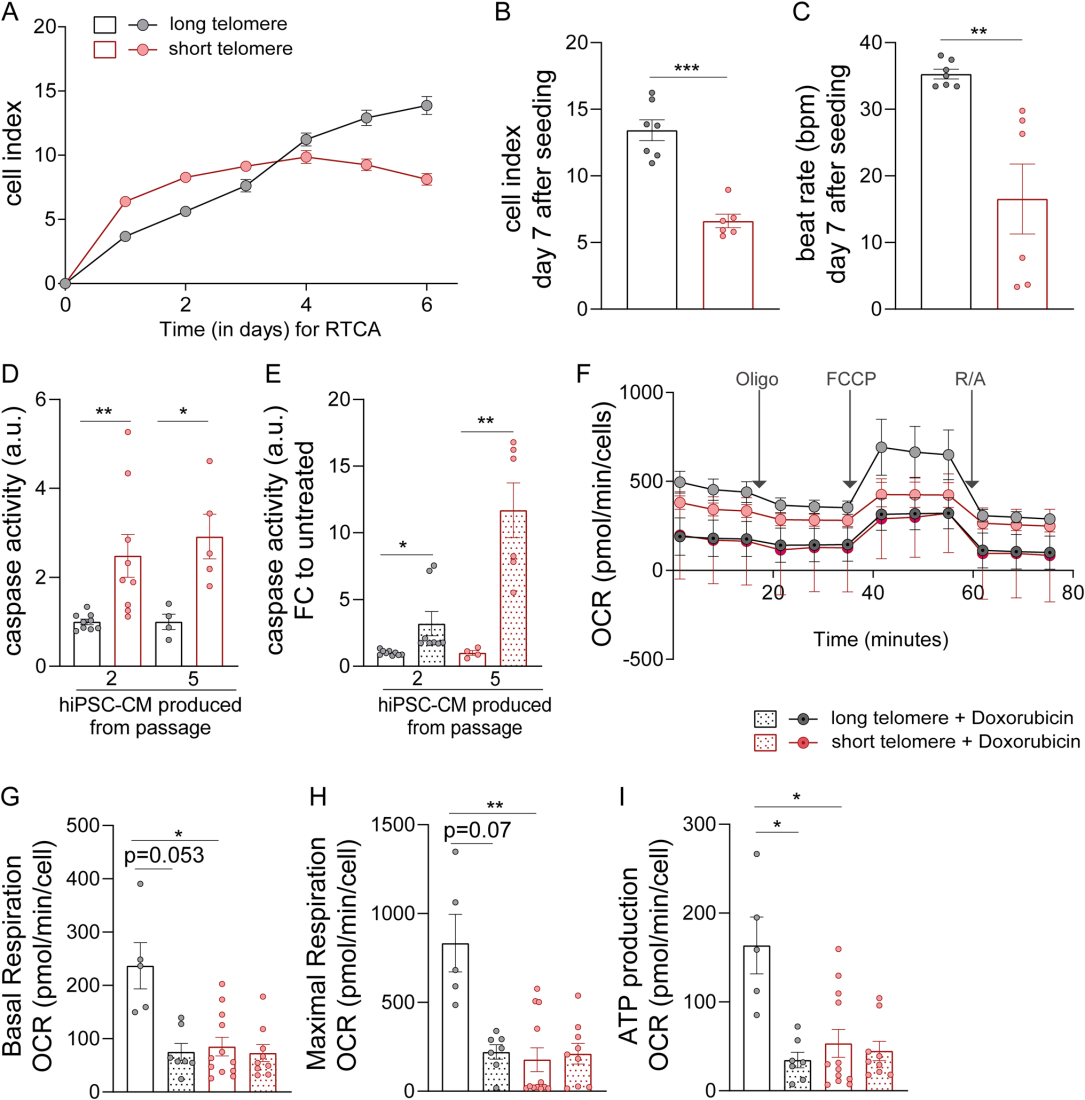
HiPSC-cardiomyocytes with short telomeres have poor cardiac health and mitochondrial function. **A** Cell Index curves indicating the initial adhesion of CRISPRi TERT hiPSC-cardiomyocytes with long (black line) and short (red line) telomeres until first 3 days followed by declining cell index of the hiPSC-cardiomyocytes with short telomeres (red line) until day 6. The curve represents the mean Cell Index value ± SD (n = 6–7 wells from one differentiation round); **B** Statistical analyses for cell index values performed at the end-point (day 7) between hiPSC-cardiomyocytes with long (black line) and short (red line) telomeres (n = 6–7 wells from one differentiation round). **C** Assessment of contractility (beats per minute, bpm) on day 7 of the RTCA analysis between hiPSC-cardiomyocytes with long (black line) and short (red line) telomeres (n = 6–7 wells from one differentiation round) with Mann–Whitney test. **D** Level of caspase activity at the basal level of CRISPRi TERT hiPSC-cardiomyocytes with long (black bar) and short telomeres (red bar) (n = 9 samples per group, triplicates from 3 independent experiments). **E** Doxorubicin (1 µM) treatment further enhances the caspase activity in CRISPRi TERT hiPSC-cardiomyocytes with shorter telomeres (+ Doxycycline) (n = 4–6 samples per group, triplicates from 2 independent differentiation experiments). **F** Analysis of mitochondrial metabolism using Seahorse XFe96 Analyzer in presence (black line) or absence (red line) of long telomeres in CRISPRi TERT hiPSC-cardiomyocytes. The CRISPRi TERT hiPSC-cardiomyocytes were further treated with Doxorubicin (1 µM, 48 h) to investigate the effect of mitochondrial function in hiPSC-cardiomyocytes with long (black line with dot) or short (red line with dot) telomeres. Oxygen consumption rate (OCR) was measured continuously at baseline and after addition of Oligomycin (2 µM), FCCP (1 µM) and R/A (0.5 µM) (n = 5–12 wells per group with 50,000 cells in each well). **G**–**I** The levels of basal respiration, maximal respiration, and ATP production before and after Doxorubicin (1 µM, 48 h) treatment. The mitochondrial metabolism reduces after doxorubicin treatment, but mitochondrial function is significantly poor in hiPSC-cardiomyocytes with short telomeres. All data are mean fold change relative to control ± SEM; hiPSC-CM = human induced pluripotent stem cell derived cardiomyocyte; Oligo = oligomycin; FCCP = carbonyl cyanide-4-phenylhydrazone; R/A = rotenone and antimycinA. *p < 0.05; **p < 0.01; ***p < 0.001; unpaired 2-tailed t-test was performed to calculate significance between 2 groups, One-way ANOVA, Kruskal–Wallis test with Dunn’s multiple comparison test

Despite a much lower efficiency of cardiomyocyte differentiation, CRISPRi TERT hiPSCs with prolonged TERT suppression can be used to generate cardiomyocytes with significantly shortened telomeres. Next, we set out to functionally characterize these cardiomyocytes with long vs short telomeres. We cultured the CRISPRi TERT hiPSC-cardiomyocytes with long and short telomeres until day 60 and first looked at cell index and contractility parameters by applying a multi electrode array (MEA) using the xCELLigence real-time cell analysis (RTCA) system. The early period of the cell index parameter is indicative of the initial phase of cell adhesion and spreading [23]. The hiPSC-cardiomyocytes with short telomeres were very fragile and exhibited poor cell adhesion compared to cardiomyocytes with longer telomeres, as determined by the declining plateau phase (Fig. 5A). Cell index at day 7 after seeding was significantly reduced in cardiomyocytes with short telomeres compared to counterparts with longer telomeres (Fig. 5B). Contractile function was also disrupted, as indicated by reduced beat rates (Fig. 5C). Furthermore, examination of the basal caspase levels indicated that cardiomyocytes with shorter telomeres exhibited elevated levels of caspase compared to controls with longer telomeres (Fig. 5D). Additional treatment with 1 µM doxorubicin, a cardiotoxic chemotherapeutic agent, triggered a prominent increase in the caspase levels of passage 5 derived hiPSC-cardiomyocytes (Fig. 5E), suggesting that short telomeres sensitized cardiomyocytes towards stress stimuli.

Since cell viability and contractility both were affected in cardiomyocytes with short telomeres, we next evaluated mitochondrial function, as a marker for cardiac maturity and function, using the Seahorse bioanalyzer. At the basal level, hiPSC-cardiomyocytes with short telomeres had lower oxygen consumption rate (OCR), basal respiration, maximal respiration and ATP production compared to cells with longer telomeres (Fig. 5F–I). Upon additional stress induced by doxorubicin, basal respiration of hiPSC-cardiomyocytes with short telomeres and long telomeres was found to be similar. Collectively, our data verified that shortened telomeres are indeed injurious for hiPSC-cardiomyocyte survival and contractile function. We also observed that if the cardiomyocytes were generated from hiPSCs with shorter telomeres, this intensified their predisposition towards cell death and mitochondrial dysfunction. Hence, hiPSC-cardiomyocytes with short telomeres could serve as excellent tools to investigate telomere length related cardiomyopathies in such an in vitro platform.

### Reversal of telomerase activity and telomeres at hiPSC stage restores cardiac function in hiPSC-cardiomyocytes

**Fig. 6:**
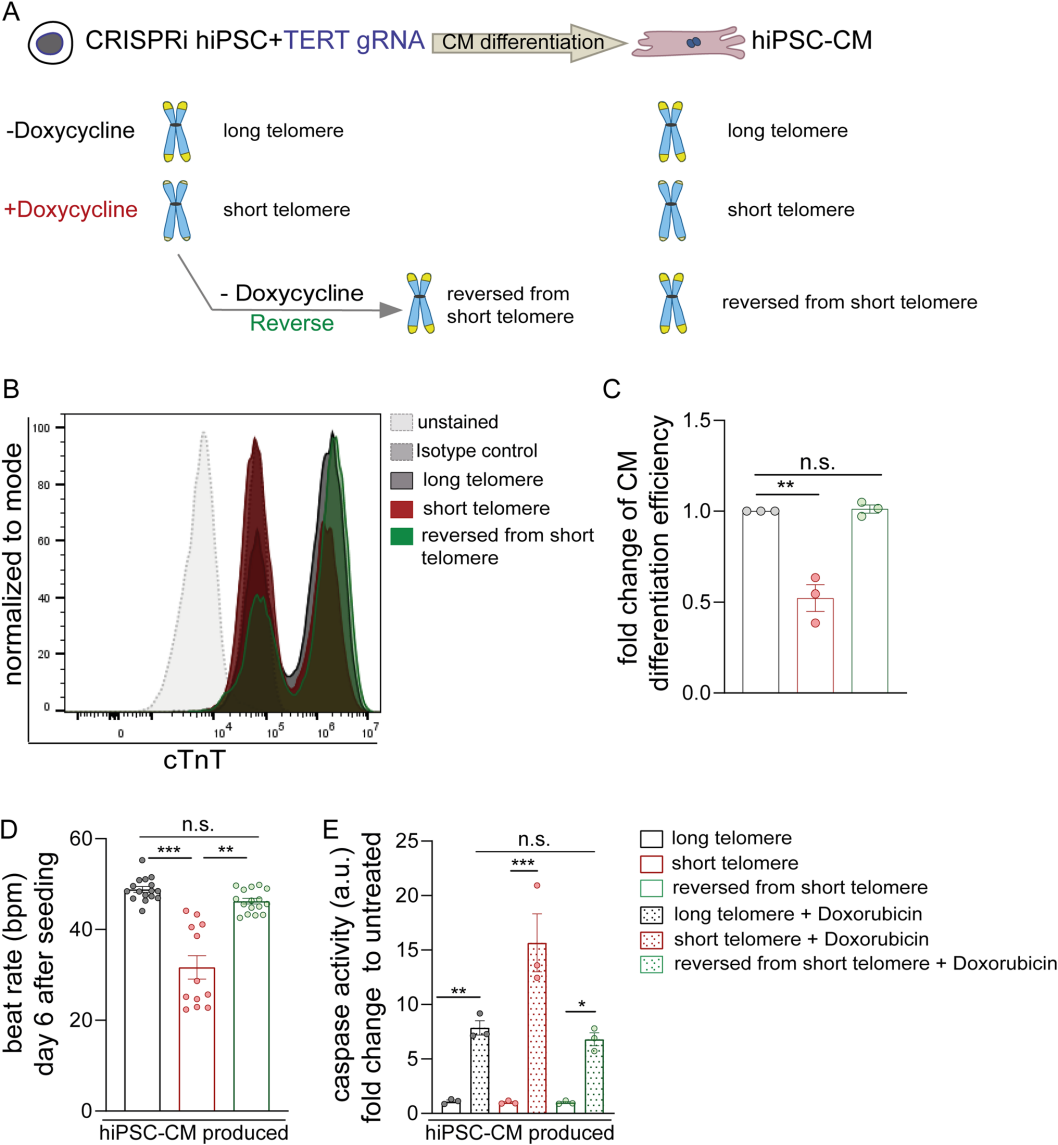
HiPSC-cardiomyocytes derived from hiPSCs with restored telomerase activity and telomere length exhibit improved function. **A** Schematic describing the reversal of the doxycycline treatment at the hiPSC stage to restore telomerase activity and telomeres and further differentiating them into hiPSC-cardiomyocytes; **B** HiPSC-cardiomyocyte differentiation efficiency measured by cTnT expression via flow cytometry; at day 12 of the cardiomyocyte differentiation protocol; from CRISPRi hiPSCs (with and without doxycycline treatment until passage 2) plus the hiPSCs after removal of doxycycline treatment for one passage to reverse the telomeres (n ≥ 5000 events per sample). Isotype controls (grey dotted line, filled) and unstained cells (light grey dotted line, filled) were used as controls. CRISPRi TERT hiPSC-cardiomyocytes arising from reverse doxycycline hiPSC group are depicted in green whereas doxycycline treated hiPSCs are depicted in red and the ones arising from control hiPSCs (− Doxycycline) are depicted in dark grey. **C** Quantification of hiPSC-cardiomyocyte differentiation efficiency amongst the CRISPRi hiPSCs with, without plus reversal of doxycycline treatment is represented in terms of fold change. All data are mean fold change relative to control ± SEM (n = 3 independent differentiation experiments); **D** Assessment of contractility (beats per minute, bpm) on day 7 of the RTCA analysis between hiPSC-cardiomyocytes with long (black), short (red) and reversed (green) telomeres (n = 12–16 wells from one CardioECR Xcelligence experiment). One-way ANOVA, Kruskal–Wallis test with Dunn’s multiple comparison test. **E** The strong apoptotic effects of Doxorubicin (1 µM) treatment as measured by caspase activity in CRISPRi TERT hiPSC-cardiomyocytes with shorter telomeres (+ Doxycycline) is diminished in the CRISPRi TERT hiPSC-cardiomyocytes with reversed telomere lengths (n = 3 samples per group, from one differentiation round). Level of caspase activity represented as fold change to the basal level of CRISPRi TERT hiPSC-cardiomyocytes with respective long (black bar), short (red bar) or reversed (green bar) telomeres. All data are mean fold change relative to control ± SEM; hiPSC-CM = human induced pluripotent stem cell derived cardiomyocyte; *p < 0.05; **p < 0.01; ***p < 0.001; unpaired 2-tailed t-test was performed to calculate significance between 2 groups, One-way ANOVA, Tukey multiple-comparisons test

In order to ascertain if these effects on cardiomyocyte function and viability are mediated by telomerase activity and telomeres, we aimed to perform the cardiomyocyte differentiation using the reversal doxycycline hiPSC group. The RNA-seq data suggested that cardiac development pathways were rescued in the reversal group (Fig. 3D, Supplemental Fig. 2C). Therefore to test this, we removed the doxycycline mediated suppression of TERT in the hiPSC stage, thereby allowing the telomerase activity and telomere lengths to revert back (as shown in Fig. 1) in these stem cells and further performed the cardiomyocyte differentiation protocol (Fig. 6A) to obtain cardiomyocytes (labelled as reversed from short telomere).

Using brightfield microscopy, we observed remarkable improvement of CRISPRi TERT reversal hiPSC group to generate cardiomyocytes (Supplemental Video 3). To confirm these observations, again cells were collected at day 12 of the hiPSC-cardiomyocyte differentiation protocol, prior to purification of cardiomyocyte cell population, and analyzed for cTnT expressing cells via flow cytometry (Fig. 6B). Strikingly, as the hiPSCs regained their telomerase activity their ability to differentiate into cardiomyocytes as determined by flow cytometry analysis, also improved (Fig. 6B, C). Thus, restoring TERT in the hiPSCs stage leads to a recovery of the hiPSC-cardiomyocyte differentiation potential. In addition to regaining the differentiation potential, the contractile function of these hiPSC-cardiomyocytes from the reversed short telomere group (green bars) was also restored as indicated by reduced beat rates (Fig. 6D, Supplemental Fig. 4A). In line with the observed impaired contractile function (Fig. 5), we monitored the sarcomeric organization in day 60 CRISPRi TERT hiPSC-cardiomyocytes by immunostaining for various myofibril markers such as cardiac Troponin I, cardiac Troponin T and F-actin filament binding dye such as Phalloidin (Supplemental Fig. 4B). The staining pattern showed an irregular myofibril arrangement in the cardiomyocytes with short telomeres which appeared better in the reverse group compared to long telomere group. Intercellular coupling and expression of appropriate ion channels are other factors which regulate contractility [24, 25]. Of note, cardiomyocytes irrespective of long or short telomeres, when seeded at high cell densities expressed Connexin43 indicating that coupling of the cells occurs normally and does not disrupt synchronous cardiac contractions (Supplemental Fig. 5A, B). Moreover, we also did not observe differences at the mRNA expression levels for various ion channels that are crucial for contractile function of cardiomyocytes (Supplemental Fig. 5B). Ultimately, the sensitivity to 1 µM doxorubicin stress, was reduced as well in the reversed group as measured by the caspase activity levels of short, long and reversed telomere hiPSC-cardiomyocytes (Fig. 6E), suggesting that indeed our CRISPRi TERT platform can be applied for telomere-related disease modelling.

## Discussion

Telomere attrition is a hallmark of aging and several age-related disorders, including CVDs. It is unclear whether telomeres continue to shorten over time in terminally differentiated cells such as cardiomyocytes, leading to disease phenotype with aging, or whether cardiomyocytes with shorter telomeres induce the malfunction in overall performance of the cardiomyocytes. TERT and TR-deficient mouse models have been instrumental to investigate the effects of telomere shortening in the context of aging and disease. However, since laboratory mice possess significantly longer telomeres (> 40 kb) compared to humans (~ 10 kb) [26] it is very cumbersome to achieve short telomere lengths to accurately recapitulate short telomere induced CVDs in vivo in these mice models [27]. Vera et al. have induced age-related neurological disorders in hiPSCs by shortening telomeres [28]. Chang and colleagues have successfully utilized the hiPSCs derived from patients with hypertrophic or dilated cardiomyopathy and further validated that patient derived cardiomyocytes in vitro also exhibit shorter telomere lengths which highlights the importance of telomeres in the context of cardiac disease progression [4].

Resetting of telomere lengths during iPSC reprogramming along with prolonged maintenance of iPSCs in culture can also cause variabilities in telomere lengths among and within iPSC clones [29]. Moreover, telomere elongation frequencies are substantially heterogeneous among independent hiPSC clones even when derived from the same donor [29, 30]. Considering that such variations might lead to erroneous interpretations, we utilized an inducible CRISPRi TERT hiPSC line allowing modulation of telomerase activity and telomere length at the hiPSC stage via doxycycline induction of reversible TERT silencing to generate an array of hiPSCs with high, intermediate and negligible telomerase activity. These categories of CRISPRi TERT hiPSCs subsequently gave rise to hiPSC-cardiomyocytes with long and short telomeres. We observed a striking decline in the production of cardiomyocytes from CRISPRi TERT hiPSCs upon TERT inhibition and progressive telomere shortening (Fig. 2C, D, Supplementary Videos 1–3), which is in line with a study by Aguado and colleagues who reported that murine iPSCs with shorter telomere lengths had reduced potency of cardiomyocyte differentiation [31]. Moreover, there is an interplay of canonical vs non-canonical Wnt signaling which orchestrates cardiomyocyte development [32] and our RNA-seq data indicates towards global changes in Wnt signaling at the hiPSC stage (Fig. 3 and Supplemental Fig. 2) as well as changes in expression of Wnt agonists at the day 12 of cardiac differentiation (Supplemental Fig. 3D) depending upon the telomerase/telomere status of the cells. It is tempting to speculate that there is an interdependence between telomerase and its unestablished non-canonical interaction with the Wnt/β-Catenin pathway. TERT is known to bind as a co-factor at the promoter region of several Wnt-dependent targets thereby acting as a transcriptional modulator of the Wnt/β-Catenin signaling cascade [33]. Our hiPSC-cardiomyocyte differentiation protocol makes use of small molecules (CHIR99021 and IWP2) leading to activation and further inhibition of the Wnt signaling. It is conceivable that such interactions play instrumental roles in orchestrating hiPSC-cardiomyocyte differentiation and further investigations are warranted. Importantly, our single cell sequencing data highlights that the hiPSCs are able to enter the endo-mesodermal lineage during differentiation irrespective of telomerase activity and telomere lengths. It is particularly the differentiation into the cardiomyocytes which is affected by the modulation of TERT in the CRISPRi TERT hiPSCs as shown by dramatic drop in differentiation efficiency (~ 60% vs. ~ 8% in cells in cells with long and short telomeres, respectively) which was then rescued by the reversal of the doxycycline treatment at the hiPSC stage.

In previous reports, Sahin et al. suggested that telomere dysfunction leads to cardiomyopathy [13] and since short telomeres are known as risk factors for CVDs [34] we proposed that hiPSC-cardiomyocytes with short telomeres should be functionally impaired or more prone to cell death in response to stress stimuli. Another recent study by Chang and colleagues showed that hyper contractility of cardiomyocytes induced telomere shortening in vitro [35], indicating towards a link between telomere length and cardiomyocyte contractility. Conversely, in our study we observed that cardiomyocytes with shorter telomere lengths had weaker cell adhesion, disorganized sarcomere alignment and reduced beating rates supporting an impact of telomere length on cardiac viability and function. This distinct finding is in line with our previously published data where we observed that shorter telomere lengths indicated towards the disease severity of patients with hypertrophic cardiomyopathy [3]. Endoplasmic reticulum membrane protein complex subunit 10 (EMC10) is not heart-specific but has been demonstrated to induce tissue repair after myocardial infarction [36] and more importantly, EMC10 promotes actin polymerization [36], a biological process that is crucial for cardiac development and maintenance. FHL2 (four-and-a-half-LIM-domain protein 2) is enriched in the heart and cardiomyocytes and has been shown to promote muscle development via interacting with β-Catenin/Wnt signaling [37]. Moreover, FHL-2 is also described to function as a specific adaptor protein linking various metabolic enzymes to titin in the sarcomeres to enable contractile function [38]. MTRNR2L8 and MTRNR2L10 are MT-RNR2-like genes with coding potential for the Humanin(HN)-like peptide and not much is known about its biological or functional role yet [39]. SFRP1 plays critical role during cardiac development by Wnt negative-feedback regulatory loop [40] and can influence the efficiency of cardiac differentiation. Moreover, hiPSC-cardiomyocytes with shorter telomere lengths were predisposed towards cell death at basal conditions and which was strongly pronounced under cardiotoxic stress conditions such as doxorubicin treatment. The negative effect of short telomeres on cardiomyogenic differentiation potential, beat rate and cell survival were rescued in the group of cardiomyocytes that were differentiated from hiPSC with reversal of telomerase activity and telomere length. This evidence from our study confirms the importance of telomere lengths in cardiac health which has been a matter of debate [41, 42]. Furthermore, using all these functional assays we validated that the CRISPRi TERT platform is extremely suitable for telomere disease modelling and potentially also for drug screening applications.

Along the lines of cardiac metabolism, we have recently demonstrated the cardioprotective role of TERT in the context of reactive oxygen species (ROS) induced mitochondrial dysfunction [10]. Thus, TERT reduction in the CRISPRi TERT hiPSCs could cause significant mitochondrial dysfunction which renders them unfit for the cardiomyocyte differentiation process. Moreover, the cardiomyocyte purification strategy using lactate media compels the mitochondria to undergo a metabolic switch. Lactate is known to induce ROS levels within the cell which ultimately confers resistance to cellular stress by a mild hormetic increase in oxidative stress [43]. Thus, it is likely that the hiPSC-cardiomyocytes with shortened telomeres suffer from high levels of ROS at basal condition which is further aggravated due to the lactate based metabolic selection. This aspect would also influence their metabolic activity which we examined using the mitostress test in seahorse bioanalyzer, for these hiPSC-cardiomyocytes with long and short telomere. Our data validates that indeed the cardiomyocytes with shorter telomeres have a poor metabolic capacity and an even lower threshold for stress stimuli as far as metabolism is concerned. In this context, telomeres were proposed to act as ROS sensors, since the G-rich telomeric repeats are particularly vulnerable to ROS-mediated oxidation [44].

The system established in this study provides the opportunity to model the human disease ex-vivo. Another advantage of our system is the presence of isogenic hiPSC-cardiomyocytes with long telomeres which can be used as controls during the screening of drugs culminating in more robust evaluation of the drugs. Our study implies that therapies targeting the proteins involved in telomere length maintenance could find potential therapeutic applications in improving the quality of cardiovascular function in heart failure patients.

## Material and methods

### Generation of stable CRISPRi TERT cell line

The inducible CRISPRi iPSC clone [15] (WTB genetic background) expressing dCas9-KRAB (KRAB domain fused at the N terminus with mCherry fluorophore) from the inducible TetO promoter (kindly shared by Conklin lab) was used to generate the CRISPRi TERT iPSC line. The gRNAs were designed to bind the parts of the TERT promoter and transfected into the CRISPRi line. The gRNA design, cloning and nucleofection was performed as described previously [15]. Briefly, TERT gRNA (5′ TTGGAAACTCGCGCCGCGAGGAGA 3′) was cloned into the pgRNA-CKB vector (73,501; Addgene) using BsmBI sites. The verified clones were transfected into CRISPRi Gen1B line using nucleofection (Human Stem Cell Nucleofector Kit 1, Lonza, #VPH-5012). Clones were selected with 10 µg/ml blasticidin for 9 days and then passaged three times. A second round of antibiotic selection was performed followed by validation of the stable clones via genotyping. This cell line was the CRISPRi TERT cell line used in this study. CRISPRi iPSCs [15] were cultured on Matrigel Growth Factor Reduced (Corning, #354,230) in mTesR (STEMCELL Technologies, #85850) and passaged when confluent with Accutase (Innovatice Cell Technologies, #AT104) in mTeSR supplemented with 10 µM Y-27632 2HCI (Selleckchem, #S1049).

### Human iPSCs culture and maintenance

CRISPRi TERT hiPSC lines were cultured on Matrigel coated cell culture plates in mTeSR full medium with supplements in an incubator at 37 °C with 5% CO_2_. After adapting from original culture conditions, the confluent hiPSCs were passaged every 5 days with Versene (Gibco, #15040-066) in mTeSR complete medium supplemented with 2 µM Thiazovivin (Selleckchem #S1459).

### Doxycycline induction for TERT modulation in hiPSCs

Daily addition of 3 µM doxycycline (doxycycline hyclate, Sigma-Aldrich, #D9891) to the hiPSC culture medium was used to induce CRISPRi targeting TERT. Cells were divided into two groups and maintained in parallel with or without doxycycline. The cells were passaged at a splitting ratio of 1:4 into the next 6-well plate for maintenance or 1:6 into 12-well plates for hiPSC-cardiomyocyte differentiation. From passage 4, the doxycycline treated cells began to exhibit delayed growth, requiring 7–8 days to become confluent. Hence, the splitting ratio was shifted to 1:3 for normal maintenance and 1:5 for cardiomyocyte differentiation in the 12-well format.

### Reversal of CRISPRi TERT modulation in hiPSCs

After the 4th passage, the doxycycline treated cells were split into two conditions, with or without continued doxycycline treatment, creating a “reversal” group of cells. For the reversal group, the cells recovered and needed only 5–6 days to become confluent and get ready for the next split. Once the cells started to exhibit normal growth patterns, the splitting ratio was shifted back to 1:4 for maintenance.

### HiPSC-cardiomyocyte differentiation and maintenance

HiPSC-cardiomyocytes were generated and maintained as described previously [45, 46] with the following modifications. Undifferentiated hiPSCs were seeded onto a Matrigel-coated 12-well cell-culture dish. The hiPSCs were maintained and passaged in mTeSR full medium supplemented with or without doxycycline (3 µM). Differentiation was induced at day 0 when CRISPRi TERT hiPSCs reached 80–90% density. On day 0, the medium was shifted to Cardio Diff medium [RPMI 1640 + GlutaMAX (Thermo Fisher Scientific, #72400021) supplemented with albumin human recombinant (Sigma-Aldrich, #A9731), L-Ascorbic acid 2-phosphate sesquimagnesium salt hydrate (Sigma-Aldrich, #A8960)] and the GSK3β inhibitor CHIR99021 (5 μM), synthesized by the Institute of Organic Chemistry, Leibniz University Hannover). After 48 h, cells were supplemented with 5 mM of the Wnt signaling inhibitor IWP2 (Peprotech, #S7085) in fresh Cardio Diff media. Following Cardio Diff medium changes were performed every 48 h. From day 8 on, the cells were fed Cardio Culture medium [RPMI 1640 + GlutaMAX supplemented with 1 × B27 with insulin (Thermo Fisher Scientific, #17504-001)], with medium changes every 2–3 days. HiPSC-cardiomyocytes were purified using metabolic selection [17] and used between day 60–90 (calculated from day 0 of differentiation) unless mentioned otherwise.

### RNA and real-time PCR

RNA was isolated using TriFast (Peqlab, #30-2030) as per the manufacturer’s instructions. Concentration of the isolated RNA was measured using Take3 Plates on a Bio-Tek plate reader (Synergy HT). For mRNA measurements, RNA (500–1000 ng) was reverse transcribed using the iScript Select cDNA Synthesis Kit (Bio-Rad, #170-8897) or Biozym cDNA synthesis kit (Biozym, #331470). A SYBR Green-based real-time PCR was performed with iQ SYBR Green mix (Bio-Rad, #172-5006CUST). The real-time PCR was performed on a ViiA7 (Applied Biosystems) using specific primer pairs (see Table below).

**Table:** 

Gene name	Species	Primer sequence (5′- > 3′)
*18s*	Human	Forward: AGTCCCTGCCCTTTGTACACA Reverse: GATCCGAGGGCCTCACTAAA
*TBP*	Human	Forward: TGCTGCGGTAATCATGAGGA Reverse: TTCACATCACAGCTCCCCAC
*hTERT*	Human	Forward: GCCTTCAAGAGCCACGTC Reverse: CCACGAACTGTCGCATGT
*hGJA1*	Human	Forward: TGGTAAGGTGAAAATGCGAGG Reverse: GCACTCAAGCTGAATCCATAGAT
*hCACNAC1Cb*	Human	Forward: ACTGGTCAGGGATGATTGGG Reverse: AGTAACTATGGCCCGAGACG
*hSCN2b*	Human	Forward: TAGCCCACCCGACTAACATC Reverse: GGTGGCACCAAAGAGAAAAA
*hKCNJ8*	Human	Forward: CCCTTTGATCATCTGCCACG Reverse: TAGGAGGTTCGTGCTTGTGT
*hHCN1a*	Human	Forward: AGAAGGAGCCGTGGGTAAAA Reverse: TCAGCAGGCAAATCTCTCCA
*hHCN1b*	Human	Forward: ACCACTACTGCAGGACTTCC Reverse: CCGACAAACATGGCATAGCA
*hKCNH2a*	Human	Forward: GACATCTTTGGGGAGCCTCT Reverse: TCGCAGGTTGAAGGTGATCT
*hKCNH2b*	Human	Forward: GCTGGATCGCTACTCAGAGT Reverse: TAGAGCGCCGTCACATACTT
*hKCND3*	Human	Forward: CCTGGGCTACACACTGAAGA Reverse: CTGCAATCGTCTTAGGCACC

### Trilineage differentiation

The CRISPRi TERT hiPSCs with and without doxycycline treatment, were differentiated into all three germ layers using STEMdiff Trilineage Differentiation Kit (STEMCELL Technologies, #05230) according to manufacturer’s guidelines.

### Immunostaining

#### Pluripotency

HiPSCs were washed three times with PBS (Merck #L182-50) and fixed with 4% formaldehyde (Roth #P733.2) for 20 min at room temperature. After washing with PBS three times, cells were permeabilised with 0.5% Tween-20 (Roth #9127), 0.1% Triton X-100 (Roth 3051.3), 0.1% IGEPAL (Sigma #9036-19-5) in TBS for 20 min. Before blocking cells were washed with TBS three times and blocked with 5% donkey serum in TBS-T (0.1% Tween-20 in TBS) for 1 h (h). Afterwards washed once with TBS-T and incubated with the primary antibody OCT4 (rabbit-anti-OCT4 C30A3, Cell signaling Technology #2840 1:400), SOX2 (rabbit-anti-SOX2 D6D9, Cell signaling Technology #3579 1:400) or NANOG (rabbit-anti-NANOG D73G4, Cell signaling Technology #4903 1:200) respectively in 1% donkey serum in TBS-T at 4 °C overnight. Cells were washed three times with TBS-T for 5–10 min, and then incubated with secondary antibody (donkey-anti-rabbit Alexa488, Invitrogen #A21206, 1:400) in 1% donkey serum in TBS-T for 1 h at room temperature. The cells were washed three times with TBS-T for 5–10 min, then stained with 1:1000 dilution of Hoechst 33342 (Thermo Fisher #H3570) in TBS for 5 min, afterwards washed twice with TBS. Images were acquired with Cytation 1 (Biotek).

#### Cardiomyocyte markers and Connexin43

HiPSC-cardiomyocytes were seeded (at low density for cardiac markers or at high density to investigate the presence of gap junction proteins) on matrigel coated glass coverslips (Marienfeld, #111580) and cultured at least for a week. The cells were washed with 1X PBS prior to fixation with 4% formaldehyde for 20 min at room temperature. After washing with PBS three times, cells were permeabilised (0.1% Triton X-100, 1% BSA in 1X PBS) for 30 min followed by blocking with (5% BSA in 1 × PBS) for 1 h. Samples were incubated with the primary antibody of rabbit- anti cardiac Troponin T (cTnT) (Abcam #ab45932, 1:500) or rabbit-anti-cardiac Troponin I (cTnI) (Abcam, #ab52862, 1:500) or rabbit-anti-Connexin43 (Abcam, #ab11370, 1:250) along with mouse-anti-Sarcomeric alpha actinin (Sigma, #a7811, 1:500) respectively in staining buffer (0.1% Triton X-100, 5% BSA in 1X PBS) overnight at 4 °C. Samples were washed three times with 1X PBS, and then incubated with secondary antibody (donkey-anti-rabbit Alexa488, Invitrogen #A21206, 1:500 or donkey-anti-mouse Alexa488, Invitrogen #A21202, 1:500 for cardiomyocyte markers and donkey-anti-rabbit Alexa680, Invitrogen #A10043, 1:500 for Connexin43 marker) in staining buffer (0.1% Triton X-100, 5% BSA in 1X PBS) for 2 h at room temperature. The cells were washed three times with 1X PBS and stained with 1:1000 dilution of Hoechst 33342 (Thermo Fisher #H3570) in PBS for 15 min, washed twice with 1X PBS before mounted the coverslips on slides with ProLong™ Gold Anti-fade Mountant (Thermo Fisher #P36930). Images were acquired with Zeiss 980 Confocal microscope.

### Flow cytometry

#### Pluripotency

CRISPRi hiPSCs were separated to single cells with Versene and 100,000 cells were suspended in FACS buffer (PBS, 1% heat-inactivated FBS, 2.5 mM EDTA). The cells were stained for 30 min at 4 °C for SSEA4 (SSEA4 Monoclonal Antibody MC-813-70, Alexa Fluor 488, eBioscience #53-8843-42), TRA-1-60 (TRA-1-60 Monoclonal Antibody, PE, eBioscience #12-8863-82) or isotype controls (mouse IgG3 FITC eBioscience #11-4742-41; mouse IgM PE R&D #IC015P). Subsequently cells were washed twice with PBS and suspended in FACS buffer. Flow cytometry was performed with Guava easyCite 5 flow cytometer (Millipore) and data was analysed using FlowJo (BD).

#### HiPSC-cardiomyocyte differentiation efficiency

HiPSC-cardiomyocytes at day 12 of differentiation protocol were harvested from 3 to 4 wells of 12-well plate, fixed with 4% formaldehyde for 15 min followed with three washing rounds in PBS and then permeabilised (0.1% Triton, 1%BSA in PBS) for 15 min. The samples were stained with cardiac Troponin T, cTnT (Abcam, ab8295, 1:500) in permeabilization buffer overnight at 4 °C. The cells were washed with PBS, followed by 2 h incubation at room temperature with 1:500 dilution of Alexa 488 (Invitrogen, #A11017) in the permeabilization buffer. The samples were washed again and re-suspended in PBS for flow cytometry measurements. Measurement was performed using CytoFLEX flow cytometer (BD) and data was analysed using FlowJo (BD).

### Telomere length measurement via qPCR (TEL-qPCR)

TEL-qPCR was performed using Telomere specific primer (T) and single copy gene specific primer (S) as described previously [47]. Telomere length of the samples is depicted as relative telomere length (T/S).

### Telomere repeat amplification protocol (TRAP)

TRAP assay was performed as described previously [10].

### Telomere quantitative fluorescence in situ hybridization (TEL-qFISH)

To determine the telomere length in the hiPSCs and hiPSC-cardiomyocytes from the various conditions, they were stained with telomere probe as described previously [10]. TEL-qFISH images were acquired using Leica SP8 or Zeiss LSM 980 confocal microscope. The analysis was performed with Image J using the Telometer Plug-in [48]. Each nuclei was manually marked within the plugin and the telomere intensities were calculated from these assigned ROIs (region of interest), as shown by dotted grey lines in the figures. Telomere length is represented as relative telomere length.

### xCELLigence RTCA

The E96 xCELLigence plates were coated with 1:100 dilution of Fibronectin (in PBS), Promocell C-43050 (50 µL per well) with 1 h incubation at 37 °C. After equilibration to 37 °C, plates were inserted into the xCELLigence station, and the base-line impedance was measured to ensure that all wells and connections were working within acceptable limits after removal of coating and addition of complete media (100 µL per well). Following harvesting and counting, hiPSC-cardiomyocytes were diluted to the correct seeding density (100 µL per well) to achieve 30,000 cells per well. Since 2 days after seeding, fresh Cardio Culture media change was performed every day (200 µL per well). Contraction data was recorded every 24 h with 30 s sweeps which were separated by an interval of 15 min. Cell index and beat rate at the 7th day (166 h) after seeding was chosen for analysis and was calculated using the built-in xCELLigence software.

### Doxorubicin treatment in hiPSC-cardiomyocytes

Doxorubicin (Sigma-Aldrich, #D1515) was applied to hiPSC-cardiomyocytes at a dose of 1 µM by adding into the Cardio Culture medium for 48 h. All the in vitro experiments were performed as three biological replicates from two to three independent rounds of hiPSC-cardiomyocyte differentiation.

### Caspase assay

Caspase assay was performed as per the manufacturer’s instructions with the Caspase-Glo 3/7 kit (Promega, #G8091). Briefly, the hiPSC-cardiomyocytes were treated with 1 µM doxorubicin in Cardio Culture medium for 48 h. After 48 h, an equal amount of caspase assay reagent (pre-warmed at room temperature) was added and further incubated for 60 min at 37 °C. Luminescence was measured using the HT Synergy (BioTek Instruments) plate reader.

### Seahorse extracellular metabolic flux assay

HiPSC-cardiomyocytes were plated at a density of 50,000 cells per well in XFe96 cell culture microplates coated with matrigel. The hiPSC-cardiomyocytes were analyzed in seahorse bioanalyzer after 48 h of 1 µM doxorubicin stress in normal maintenance medium. The XF Cell Mito Stress Test Kit (Agilent, #103015-100) was used as per the manufacturer’s protocol to study the mitochondrial function of hiPSC-cardiomyocytes under basal and doxorubicin stress conditions. The bioenergetics was measured with the Seahorse XFe96 Analyzer. Media was aspirated, cells were washed twice and finally replaced with 180 µL assay medium (Agilent; #103575-100) and pre-equilibrated for 1 h at 37 °C. Baseline OCR measurements were acquired followed by injection of 2 µM oligomycin (Oligo), 1 µM FCCP (Trifluoromethoxy carbonylcyanide phenylhydrazone, Carbonyl cyanide 4-(trifluoromethoxy)phenylhydrazone) and finally 0.5 µM rotenone and antimycin A (R/A). Each injection was followed by three OCR measurements. After the seahorse run hiPSC-cardiomyocytes were stained with Hoechst 33342 (1:1000, Thermo fisher; #62249) and imaged using Cytation 1 (Biotek). The cell numbers were used to normalize the seahorse data and 5–12 wells per group were analyzed in this metabolic assay.

### Single cell sequencing

For each sample, cell suspension was prepared using mild digestion to maintain viability of the sample followed by filtration to obtain single cell suspension. More than 6000 cells were utilized from each condition for the single cell sequencing.

Library preparation for single cell mRNA-Seq analysis was performed according to the Chromium Single Cell 3′ Reagent Kit v3 User Guide (Manual Part Number CG000183 Rev A; 10 × Genomics). According to the protocol a given 1.6-fold excess of cells was loaded to the 10 × controller in order to reach a target number of 7000 cells per sample. Fragment length distribution of generated libraries was monitored using ‘Bioanalyzer High Sensitivity DNA Assay’ (5067–4626; Agilent Technologies). Quantification of libraries was performed by use of the ‘Qubit® dsDNA HS Assay Kit’ (Q32854; ThermoFisher Scientific).

Equal molar amounts of three libraries were pooled, denatured with NaOH, and were finally diluted to 1.8 pM according to the Denature and Dilute Libraries Guide (Document # 15048776 v02; Illumina). 1.3 ml of denatured pool was sequenced on an Illumina NextSeq 550 sequencer using a High Output Flowcell for 76 cycles and 400 million reads (#20024906; Illumina). Samples were sequenced according to the following settings: 28 bp as sequence read 1; 56 bp as sequence read 2; 8 bp as index read 1; no index read 2.

The proprietary 10 × Genomics CellRanger pipeline (v3.0.2) was used with default parameters except for the setting of expected cells (7000-cells). After default CellRanger quality filtering, transcriptomes from 6109, 9045 and 7716 cells were kept from day 12 of the hiPSC-cardiomyocyte differentiation pool with long, intermediate and short telomeres, respectively.

### Single cell RNA sequencing analysis

Initially, the mkfastq command was used to demultiplex the data from the raw BCL sequencer images, given the supplied sample sheet with the 10X barcodes. This step resulted in standard FASTQ files and 10X specific metadata. Next, CellRanger was used to align read data to the reference genome provided by 10X Genomics (Human reference dataset 3.0.0; November 19, 2018; GRCh38) using the aligner STAR, counting aligned reads per gene, and calculating clustering and summary statistics.

Downstream analysis from normalization to cell clustering were performed utilizing SCANPY (v.1.7.1) [49] integrated in Galaxy platform [50]. In order to get a high quality and high-resolution map of cell clusters, we included additional quality control steps (pp.calculate_qc_metrics and pp.normalize_total). In SCANPY the minimum number of genes expressed for a cell was set to 1000; the maximum percent of mitochondria genes was set to 10, which left us with 4539, 7318 and 5730 cells from the samples with long, intermediate and short telomeres, respectively from day 12 of the hiPSC differentiation pool for the single cell RNA sequencing analysis.

The Loupe Cell Browser (version 6.0) from 10 × was used to visualize and explore the cell clusters, which were derived by utilizing a dimension reduction technique UMAP [51] and clustering algorithm Louvain [52]. This process resulted in 10 clusters, which were then manually annotated using six cell-type-specific canonical gene markers.

### Bulk RNA sequencing and analysis

#### Sample preparation and RNA isolation

CRISPRi TERT hiPSCs were subjected to no doxycycline (C) or doxycycline induction for 2 passages (P2), 5 passages (P5) and 2 weeks reversal (R). Samples were harvested in QIAzol (Qiagen, 79306) and RNA was isolated using miRNeasy Mini Kit (Qiagen, 217004) according to the manufacturer’s guidelines. RNA quality was checked using Bioanalyzer and four samples from each condition were used for bulk RNA sequencing using sequencing services at Novogene GmbH.

### Library generation, quality control, and quantification

For library preparation, polyA-tail enrichment was performed by isolating the mRNA with magnetic beads of oligos d(T)25. Subsequently, mRNA was randomly fragmented and cDNA synthesis was done using random hexamers. The second chain was synthesized using Nick translation in Illumina buffer containing dNTPs, RNAseH and polymerase I from *E. coli*. The resulting products were purified and ligated with adapter. Fragments of the appropriate size were enriched by PCR and P5 and P7 indexed primers were introduced before the final products were purified. The library was checked with Qubit 2.0 and real time PCR for quantification and bioanalyzer Agilent 2100 for size distribution detection.

### Library denaturation and Sequencing run

Qualified libraries were pooled and loaded on a Novaseq X plus sequencer with paired-end 150 bp. Mapping of raw reads to the reference genome (GRCh38) was performed using hisat2 (v 2.0.5) and featureCounts (v 1.5.0-p3) was used for gene-level quantification of mapped reads.

### Raw data processing and quality control

The R package DESeq2 (v 1.42.0) was used to calculate differentially expressed genes between the treatment and control groups using a single-factor design based on raw gene counts. g:Profiler web tool was used to perform functional overrepresentation analysis of significantly deregulated genes. Selected results were plotted as bubble plots using ggplot2 (v 3.4.4) package in R. Clustered and unclustered heatmaps of normalized counts of selected genes were plotted using pheatmap package (v. 1.0.12).

### Statistics

All the data were analyzed with GraphPad Prism software (version 8.0) and are presented as mean ± SEM. All the data sets were tested for normality of distribution using the Shapiro–Wilks test (threshold P < 0.05). For normally distributed data, values shown are mean ± SEM. For comparison of 2 groups, an unpaired students t-test was conducted and of ≥ 3 groups, a one-way-ANOVA was conducted with a post hoc Tukey test multiple comparison test. For non-normally distributed data a nonparametric test was used to test for significance between different groups. A Mann–Whitney test was performed when comparing two groups. A Kruskal–Wallis test was used when comparing multiple groups (more than two) followed by a Dunn's multiple test comparison.

### Supplementary Information

Below is the link to the electronic supplementary material.

Supplementary file1 (TIF 124457 KB)

Supplementary file2 (TIF 77230 KB)

Supplementary file3 (TIF 98530 KB)

Supplementary file4 (TIF 111041 KB)

Supplementary file5 (TIF 98160 KB)

Supplementary file6 (XLSX 33 KB)

Supplementary file7 (XLSX 11 KB)

Supplementary file8 (XLSX 20 KB)

Supplementary file9 (XLSX 3062 KB)

Supplementary file10 (XLSX 3107 KB)

Supplementary file11 (MP4 4969 KB)

Supplementary file12 (MP4 7719 KB)

Supplementary file13 (MP4 98782 KB)

Supplementary file14 (PDF 458 KB)

## Data Availability

The scRNA seq dataset generated and analysed during the current study is available on the Gene Expression Omnibus (GEO) repository (GSE239887). The bulk RNA sequencing data can be accessed at the GEO repository (GSE260618).
