# Supplementary material for: Telomerase is essential for cardiac differentiation and sustained metabolism of human cardiomyocytes
Source: Cell Mol Life Sci. 2024 Apr 24;81(1):196. doi: 10.1007/s00018-024-05239-7 (PMC11043037; doi:10.1007/s00018-024-05239-7)
Supplement: Supplementary file 14 — Supplementary file14 (PDF 458 KB) [file 18_2024_5239_MOESM14_ESM.pdf]

## Supplementary Information

# Telomerase is essential for cardiac differentiation and sustained metabolism of human cardiomyocytes


Shambhabi Chatterjee[1,2,3], Megan Leach-Mehrwald[1], Cheng-Kai Huang[1], Ke Xiao[1,3], Maximilian Fuchs[3], Mandy Otto[3], Dongchao Lu[1,2], Vinh Dang[4], Thomas Winkler[4], Cynthia E. Dunbar[4], Thomas Thum[1,2] and Christian Bär[1,2,3#]

[1] Institute of Molecular and Translational Therapeutic Strategies (IMTTS), Hannover Medical School, Hannover, Germany.

[2] Center of Translational Regenerative Medicine, Hannover Medical School, Hannover, Germany

[3] Fraunhofer Institute for Toxicology and Experimental Medicine, Hannover, Germany

[4] Translational Stem Cell Biology Branch, National Heart, Lung, and Blood Institute, National Institutes of Health, Bethesda, Maryland, USA.

#Correspondence: Christian Bär, PhD

Hannover Medical School, Institute of Molecular and Translational Therapeutic Strategies

Tel: 0049-511-532-2883

Email: baer.christian@mh-hannover.de

Supplemental Figure 1

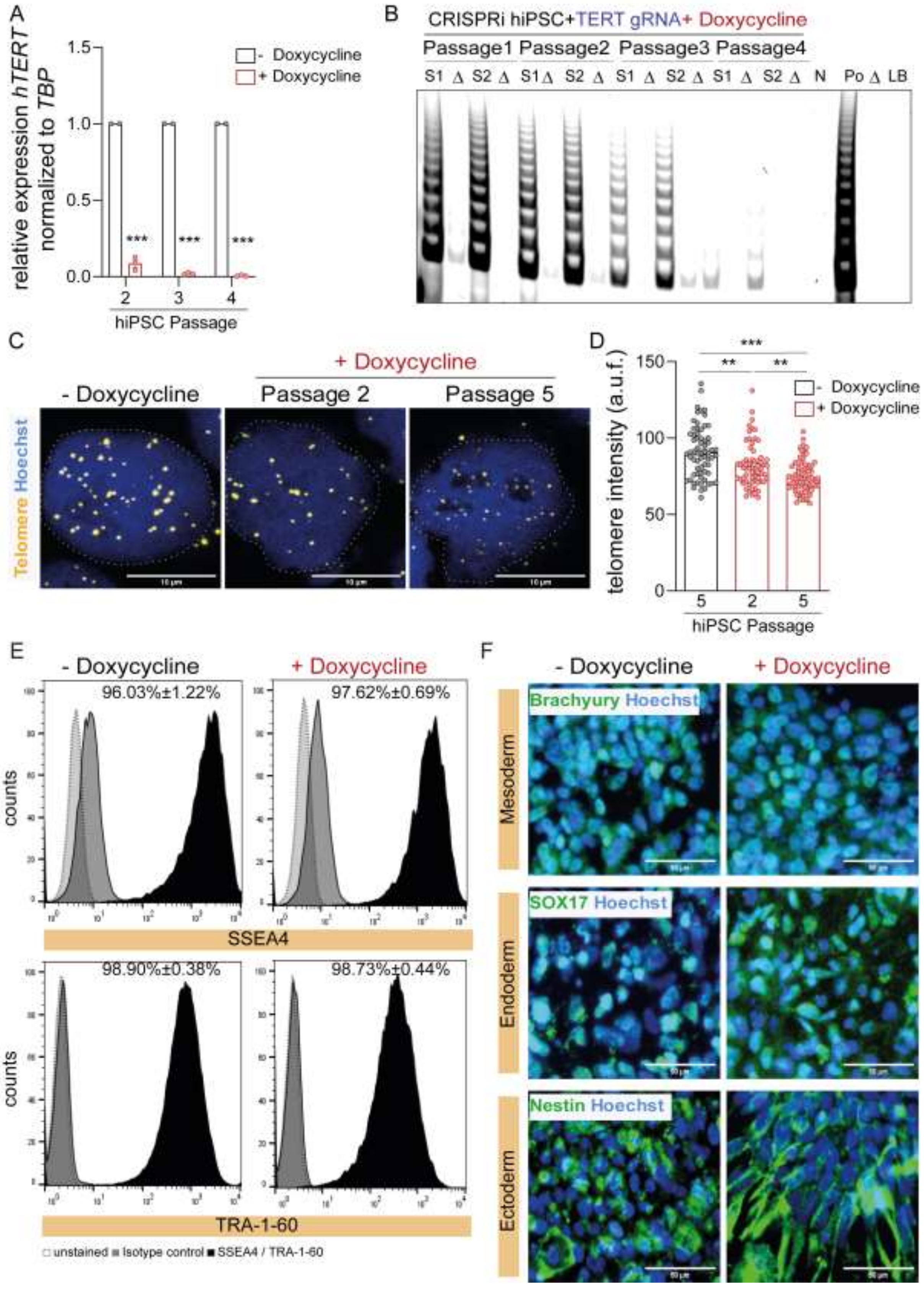

**Supplemental Figure 1| Induction of telomerase modulation in CRISPRi TERT hiPSCs does not influence pluripotency.** (A) The mRNA expression of *hTERT* during doxycycline treatment of CRISPRi TERT hiPSCs (red bars) from passage 2 to 4 compared to untreated (- Doxycycline, black bar) (n=4/4/3). (B) Telomerase activity in cell lysates of CRISPRi TERT hiPSCs under doxycycline treatment gradually diminishes from passage 1 to 4. (n=200,000 cells per group), S indicates sample; $\Delta$ indicates heat inactivated cell lysate; N indicates cell lysate of TRAP negative sample (HUVEC); Po indicates cell lysate of TRAP positive sample (hiPSCs); LB indicates lysis buffer. (C-D) Representative images and quantification performed for CRISPRi TERT hiPSCs at passage 2 and 5 with doxycycline compared to controls (- Doxycycline) using TEL-qFISH. Subsequent shortening of telomere lengths is observed upon doxycycline-mediated CRISPRi induction from passage 2 to passage 5 (red bar) (n≥66 nuclei per group). Scale bar indicates 10 µm; The dotted black line indicates the nuclei region used for qFISH analysis. hiPSCs= human induced pluripotent stem cells; *** $p<0.001$; One-way ANOVA, Kruskal-Wallis test with Dunn's multiple comparison test. (E) Flow cytometry analysis of SSEA4 and TRA-1-60 (filled black) with and without telomerase modulation CRISPRi TERT hiPSCs using doxycycline. Unstained cells (filled gray) and isotype controls (filled dark gray) were used as controls. (F) Trilineage differentiation of CRISPRi TERT hiPSCs in the presence and absence of doxycycline treatment to the three germ layers as analyzed by immunofluorescence staining. Scale bar indicates 50 µm.

Supplemental Figure 2

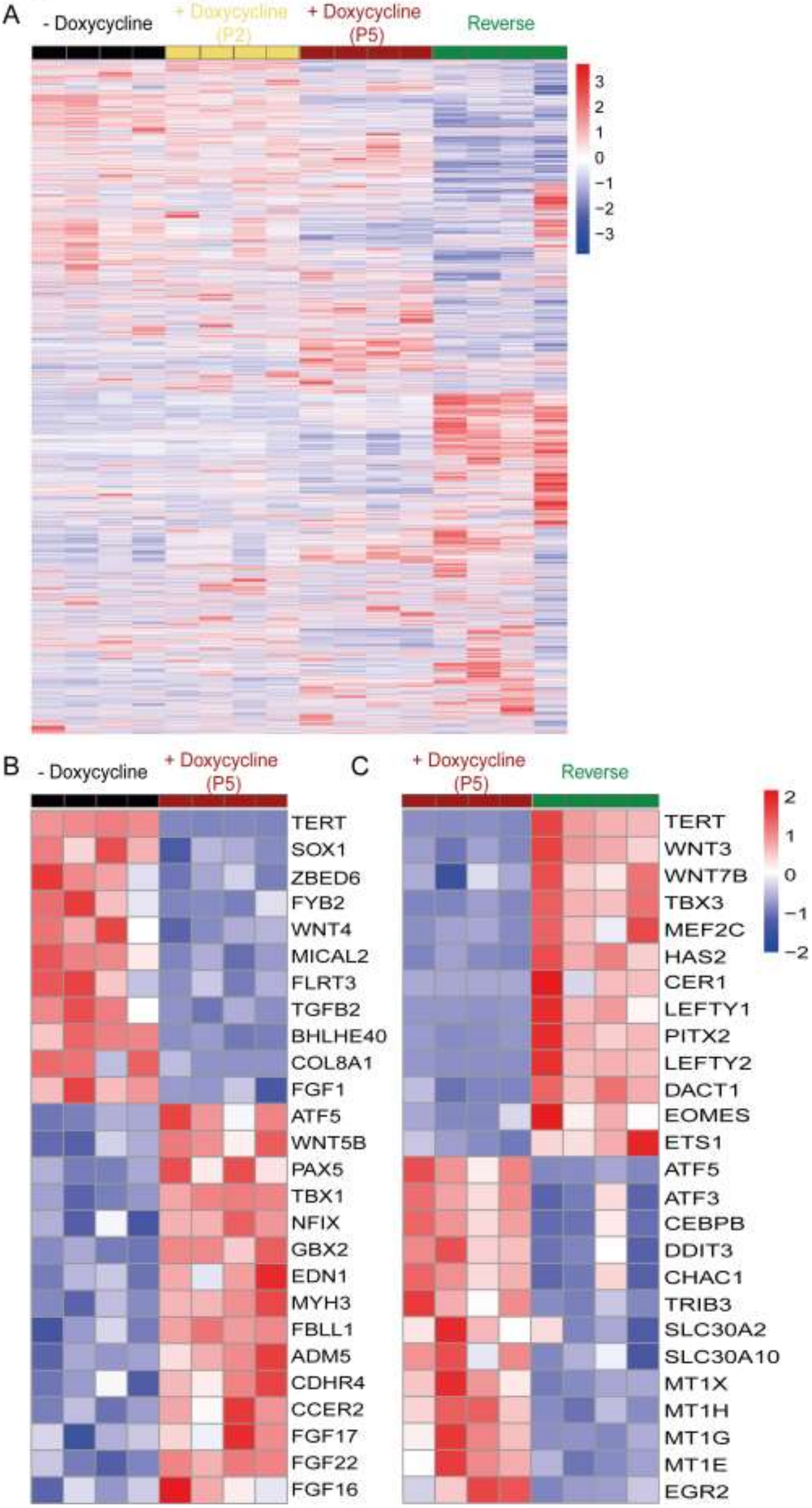

**Supplemental Figure 2| Heirarchical clustering of CRISPRi TERT hiPSCs RNA-sequencing data.** (A) Heat map of RNA-Seq data for top 2000 differentially regulated genes from the CRISPRi TERT hiPSCs treated with doxycycline for 2 passages (P2) and 5 passages (P5) compared to untreated (C) and doxycycline reverse for 2 weeks after P5 (R) (blue indicates down-regulated genes, red indicates up-regulated genes). (B) Heatmap shows significantly different mRNA expressions between the two groups: C and P5 for genes related to developmental pathways (Supplemental Table 4 list of DEGs between C and P5). (C) Heatmap shows selected up and downregulated genes after reversal of doxycycline treatment (R) compared to P5 group. (Supplemental Table 5 list of DEGs between R and P5)

Supplemental Figure 3

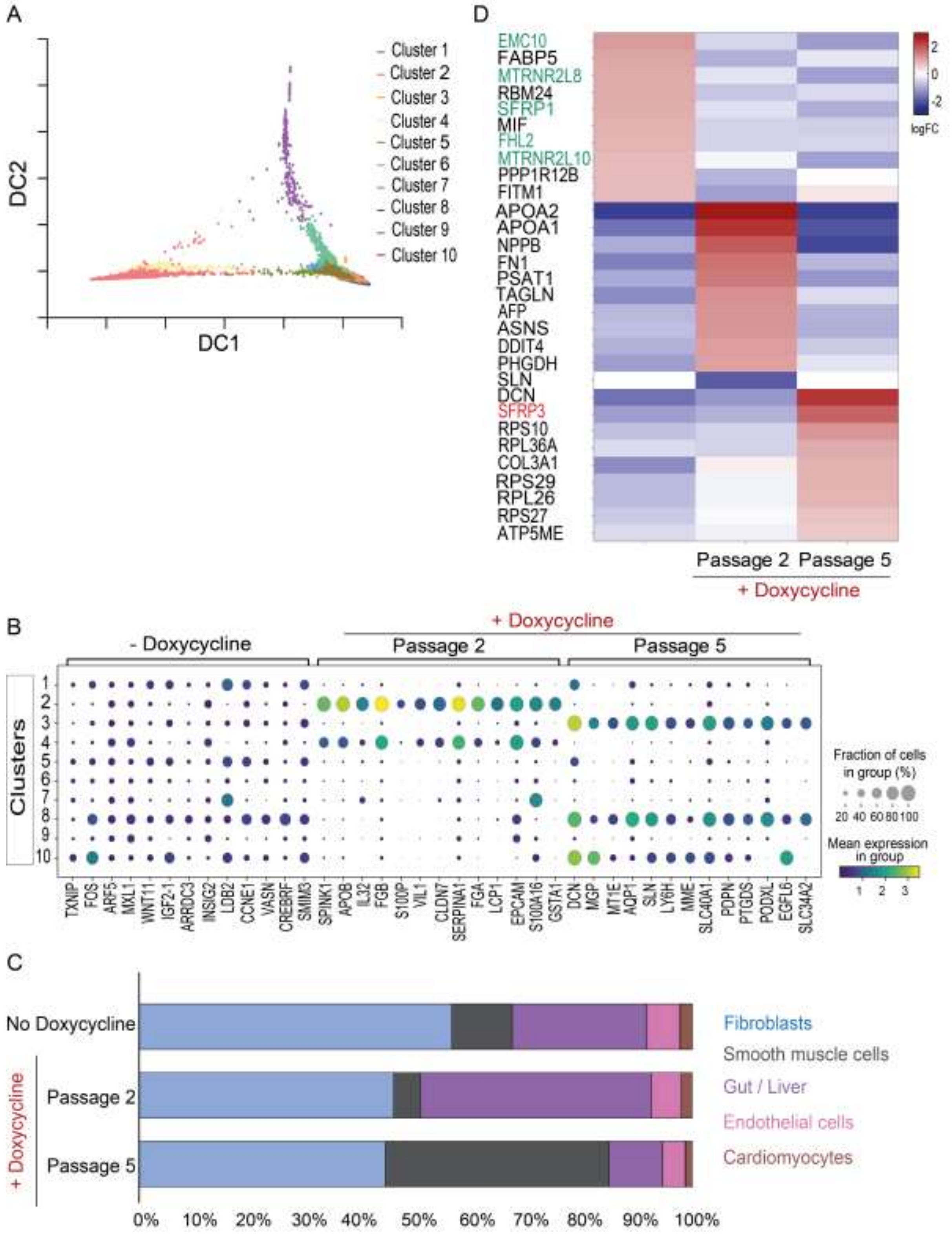


**Supplemental Figure 3| Clustering analysis of single cell RNA-sequencing data.**

(A) Expression profiles of genes that are highly and specifically enriched in the

individual clusters. The dot plot indicates specific gene sets that are enhanced (cluster 2 and 4 and clusters 3, 8 and 10) due to telomere shortening. Bar on the right displays the percentage of total dataset represented in every cluster, showing the abundance of each cell type found by clustering analysis. (B) Diffusion map showing the two differential trajectories arising during the mesodermal differentiation. (C) Appearance of specific clusters arising from the differentiation of the various doxycycline induced CRISPRi cells. (D) Heatmap: top 10 most differentially expressed genes between hiPSC-cardiomyocytes with long (-Doxycycline) and short (+Doxycycline) telomeres. Genes that progressively get downregulated in cardiomyocytes as the telomeres shorten are highlighted in green. SFRP3 gene highlighted in red indicates the isoform of SFRP1 which shows reciprocal trend with telomere shortening conditions.

Supplemental Figure 4

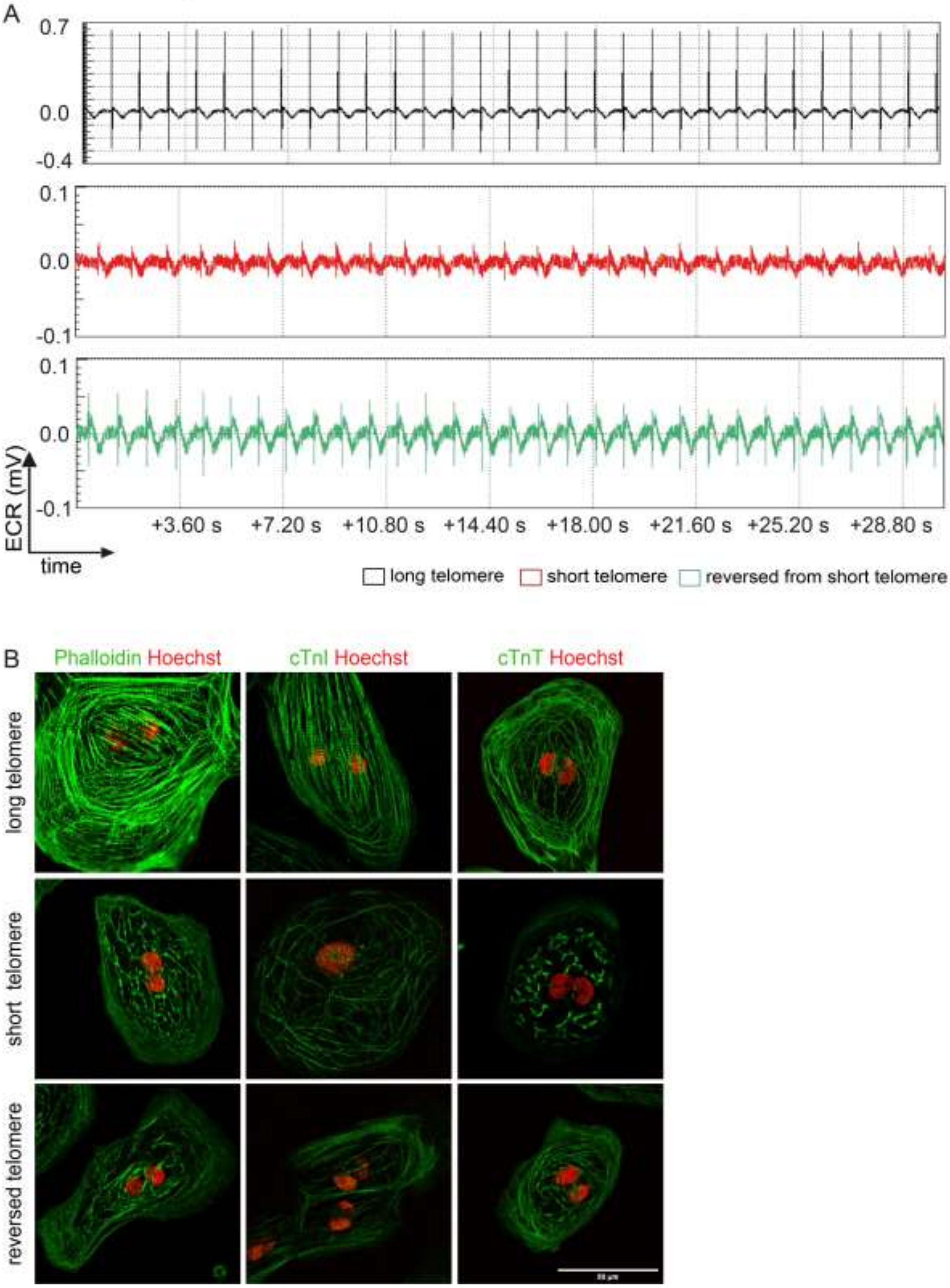

**Supplemental Figure 4| Impedance derived contractile function of CRISPRi TERT hiPSC-cardiomyocytes.** (A) Recording of field potential signal traces (30 s) from a representative well of CRISPRi TERT hiPSC-cardiomyocytes with long telomeres (-Doxycycline, black traces) and short telomere (+ Doxycycline, red traces) and reversed from short telomere (green traces) on day 7 after seeding in the xCELLigence RTCA system indicating changes in beat rate. (B) Single cells from CRISPRi TERT hiPSC-cardiomyocytes at day 60 with long telomeres, short telomeres and reversed from short telomere stained with various markers (Phalloidin, cardiac Troponin I (cTnI) and cardiac Troponin T (cTnT)) in green to highlight the sarcomere organization within these cells. Nuclei is stained with Hoechst (red). Scale bar 50 μm.

Supplemental Figure 5

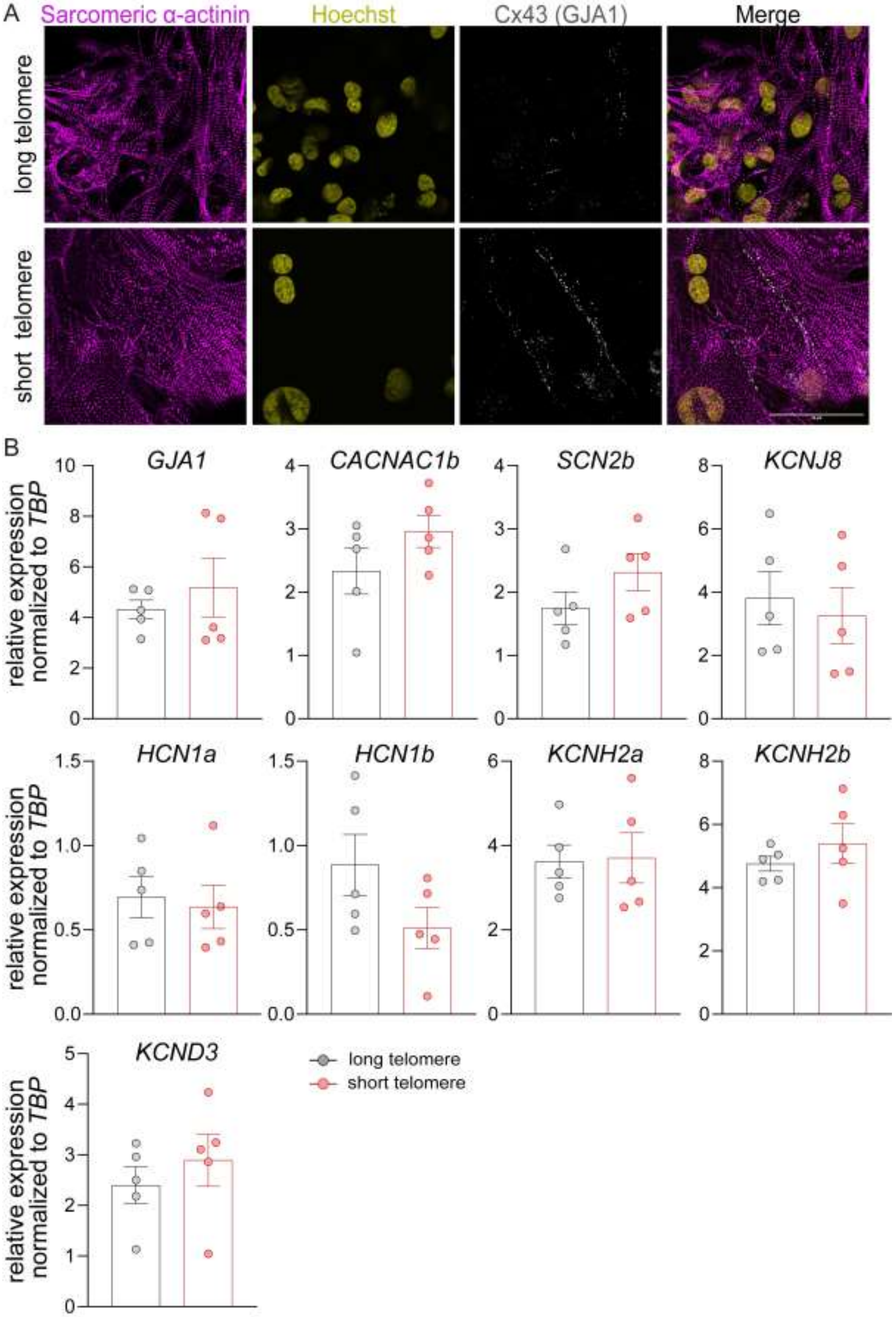

**Supplemental Figure 5| Intercellular coupling and cell excitability in CRISPRi TERT hiPSC-cardiomyocytes.** (A) Representative images from monolayer of CRISPRi TERT hiPSC-cardiomyocytes at day 60 with long telomeres and short telomeres stained with gap junction marker Connexin43 (Cx43) in gray along with sarcomeric alpha actinin (sarcomeric α-actinin) in magenta indicating intercellular coupling within these cardiomyocytes. Nuclei is stained with Hoechst (yellow). Scale bar 50 µm. (B) Levels of mRNA expression of gap junction (*GJA1*) and several ion channels (*CACNAC1b*, *SCN2b*, *KCNJ8*, *HCN1a*, *HCN1b*, *KCNH2a*, *KCNH2b*, *KCND3*) in CRISPRi TERT hiPSC-cardiomyocytes at day 60 with long telomeres (-Doxycycline) or short telomere (+ Doxycycline). (n= 5 differentiation rounds / group) No significant difference was found by performing unpaired 2-tailed t-test between the 2 groups, except for *GJA1* where Mann–Whitney test was applied

**Supplemental Tables**

Table 1 – list of top 2000 expressed genes from various CRISPRi TERT hiPSC treatment groups used in bulk RNA-seq

Table 2 – full gprofiler list of Gene ontology enrichment pathways between C and P5 group.

Table 3 – full gprofiler list of Gene ontology enrichment pathways between P5 and R group.

Table 4 –unfiltered list of all DEGs from CRISPRi TERT hiPSCs C vs P5

Table 5 –unfiltered list of all DEGs from CRISPRi TERT hiPSCs R vs P5

**Supplemental videos**

Video 1 – recording of spontaneously beating layer of cardiomyocytes differentiated from CRISPRi TERT hiPSCs with long telomere at day 12 of differentiation.

Video 2 – recording of cardiomyocytes differentiated from CRISPRi TERT hiPSCs with short telomeres indicating very less cardiomyocyte regions at day 12 of differentiation.

Video 3 – recording of cardiomyocytes differentiated from CRISPRi TERT hiPSCs with telomeres reversed from short telomere condition indicating improved cardiomyocyte differentiation potential at day 12 from start of differentiation.
